# Unusual free oligosaccharides in human bovine and caprine milk

**DOI:** 10.1038/s41598-022-15140-7

**Published:** 2022-06-24

**Authors:** Wei-Chien Weng, Hung-En Liao, Shih-Pei Huang, Shang-Ting Tsai, Hsu-Chen Hsu, Chia Yen Liew, Veeranjaneyulu Gannedi, Shang-Cheng Hung, Chi-Kung Ni

**Affiliations:** 1 Institute of Atomic and Molecular Sciences, Academia Sinica, Taipei, 10617 Taiwan; 2 Molecular Science and Technology, International Graduate Program, Academia Sinica and National Tsing Hua University, Hsinchu, 30013 Taiwan; 3 Department of Applied Chemistry, National Yang Ming Chiao Tung University, Hsinchu, 300 Taiwan; 4 International Graduate Program of Molecular Science and Technology, National Taiwan University and Taiwan International Graduate Program of Molecular Science and Technology, Academia Sinica, Taipei, 10617 Taiwan; 5 Genomic Research Center, Academia Sinica, Taipei, 11529 Taiwan

**Keywords:** Mass spectrometry, Glycobiology, Dietary carbohydrates

## Abstract

Free oligosaccharides are abundant macronutrients in milk and involved in prebiotic functions and antiadhesive binding of viruses and pathogenic bacteria to colonocytes. Despite the importance of these oligosaccharides, structural determination of oligosaccharides is challenging, and milk oligosaccharide biosynthetic pathways remain unclear. Oligosaccharide structures are conventionally determined using a combination of chemical reactions, exoglycosidase digestion, nuclear magnetic resonance spectroscopy, and mass spectrometry. Most reported free oligosaccharides are highly abundant and have lactose at the reducing end, and current oligosaccharide biosynthetic pathways in human milk are proposed based on these oligosaccharides. In this study, a new mass spectrometry technique, which can identify linkages, anomericities, and stereoisomers, was applied to determine the structures of free oligosaccharides in human, bovine, and caprine milk. Oligosaccharides that do not follow the current biosynthetic pathways and are not synthesized by any discovered enzymes were found, indicating the existence of undiscovered biosynthetic pathways and enzymes.

## Introduction

Free oligosaccharides are among the most abundant macronutrients in milk^1,2^. Milk oligosaccharides are a class of indigestible carbohydrates. The majority of free oligosaccharides consumed by infants from their mother’s milk are not digested and absorbed within the small intestine, and most of these oligosaccharides reach the colon of infants. The biological functions of free oligosaccharides present in milk have been of scientific interest for many years. Recent studies have suggested that milk oligosaccharides are involved in prebiotic functions and the antiadhesive binding of viruses and pathogenic bacteria to colonocytes^2,3^.

Most free milk oligosaccharides contain lactose at the reducing end, and this disaccharide is assumed to be the initial substrate in biosynthesis. α-Lactalbumin, which is only found in lactating mammary glands and milk, plays a crucial role in the biosynthesis of oligosaccharides in milk. The biosynthesis starts from lactose, which is synthesized from UDP-Gal and glucose through a complex consisting of β-1,4-galactosyltransferase I and α-lactalbumin. The further glycosylation of lactose by glycosyltransferases produces various oligosaccharides^4–9^.

Although the structures of free oligosaccharides present in milk have been widely studied^10–15^, the non-template-based biosynthesis of oligosaccharides and the large number of oligosaccharide isomers make structural identification challenging even today. The full structures of oligosaccharides were conventionally determined through wet chemistry^16–20^ and nuclear magnetic resonance spectroscopy (NMR)^21–26^. However, these methods are time consuming and require large amount of samples. These methods are suitable only for highly abundant oligosaccharides. The sensitivity of mass spectrometry is three to four orders of magnitude higher than that of wet chemistry and NMR. Mass spectrometry has been widely used for the structural determination of oligosaccharides^27–29^. However, only part of the structures of oligosaccharides, including compositions, sequences, and linkage positions, can be identified through conventional mass spectrometry. Conventional mass spectrometry does not provide information on anomericity (differentiation of the α and β anomeric configuration of glycosidic bonds) and monosaccharide stereoisomers (e.g., differentiation of glucose, galactose, and mannose or N-acetylglucosamine and N-acetylgalactosamine). Although structures obtained through conventional mass spectrometry can be further identified according to possible biosynthetic pathways, using exoglycosidase digestion, or through comparison with oligosaccharide standards from an oligosaccharide mass spectrum library^30–34^, the results obtained are limited by current knowledge on biosynthetic pathways, the available enzymes, and existing oligosaccharide standards in libraries, prohibiting the discovery of new oligosaccharides.

Recently, a new mass spectrometry method, namely logically derived sequence (LODES) multistage tandem mass spectrometry (MS^n^) was developed for the full structural determination of oligosaccharides^35–38^. This method can be used to determine oligosaccharide structures including linkage positions, anomericities, and monosaccharide stereoisomers. The method has been validated using various oligosaccharide standards^35–41^, it has exhibited high sensitivity and can be combined with high-performance liquid chromatography (HPLC) for the online structural identification of small oligosaccharides. In this study, we applied LODES/MS^n^ for the structural determination of free oligosaccharides extracted from human, bovine, and caprine milk. We focused on the trisaccharides and tetrasaccharides which have not been reported before. These unreported oligosaccharides have low abundance that the structures are difficult to be determined using NMR or enzyme digestion but are suitable for mass spectrometry. Many of the newly discovered oligosaccharides do not follow the current biosynthetic pathways of free oligosaccharides in human milk and are not synthesized by any discovered enzymes, pointing toward the existence of further undiscovered biosynthetic pathways and enzymes. The present study compared free oligosaccharide biosynthetic pathways in human milk versus bovine milk and proposed new biosynthetic pathways. No undiscovered fucosylated and sialylated trisaccharides and tetrasaccharides were found, thus the fucosylated and sialylated oligosaccharides are not reported in this study.

## Results

Free oligosaccharides extracted from milk were separated into several groups by using a size exclusion column followed by HPLC with an amide-80 column. Each fraction of eluents obtained after amide-80 column separation was further separated into individual isomer through another HPLC with a porous graphitic carbon (PGC) column. Eluents obtained from the PGC column were transported into a linear ion trap mass spectrometer for structural determination by using LODES/MS^n^. Only neutral trisaccharides extracted from human, bovine, and caprine milk and neutral tetrasaccharides extracted from human and bovine milk consisting glucose, galactose, mannose, N-acetylglucosamine, and N-acetylgalactosamine were examined in this study.

### Trisaccharides

**Figure 1:**
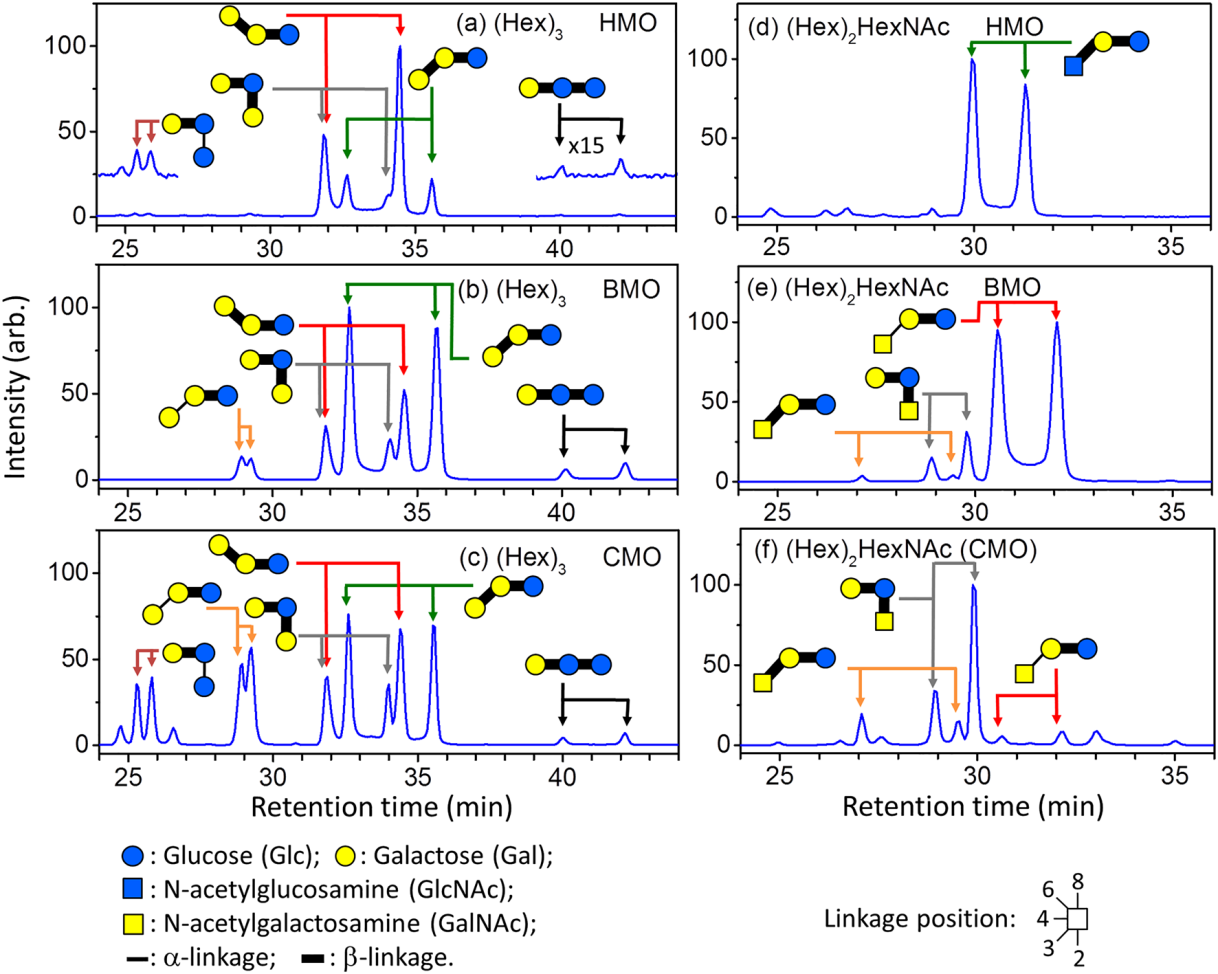
PGC chromatograms of oligosaccharides separated using a PGC column from a fraction of eluents separated using an amide-80 column for human milk oligosaccharides (HMO), bovine milk oligosaccharides (BMO), or caprine milk oligosaccharides (CMO). (**a–c**) ion *m/z* 527 (Hex)_3_ sodium adducts, and (**d–f**) ion *m/z* 568 (Hex)_2_HexNAc sodium adducts.

Figure 1a–c, and d–f illustrate the chromatograms of ions *m/z* 527 [sodium adducts of (Hex)_3_] and *m/z* 568 [sodium adducts of (Hex)_2_HexNAc], respectively. Each chromatogram represents oligosaccharide separation performed using a PGC column from a fraction of eluents separated using the amide-80 column for human milk oligosaccharides (HMO), bovine milk oligosaccharides (BMO), or caprine milk oligosaccharides (CMO). The fraction of eluents separated by the amide-80 column was chosen such that all oligosaccharides identified in this study could be shown in a single chromatogram for compact display in Fig. 1. Fractions consisting of fewer oligosaccharides are not shown. Therefore, the relative intensity shown in the chromatogram in Fig. 1 does not represent the relative abundance of these oligosaccharides in milk.

Notably, oligosaccharides extracted from milk in this study were not reduced at the reducing end. They were intact, and each oligosaccharide had two anomers (α and β of the sugar at the reducing end), which coexisted in the solution. The PGC column could separate these two anomers for most oligosaccharides, resulting in two peaks for each oligosaccharide in the chromatogram. Although the chromatogram became complicated when oligosaccharides were not reduced, the use of intact oligosaccharides has several advantages. First, no loss of sample during the reduction process; this increased the sensitivity for detecting less abundant oligosaccharides. Second, no products generated from potential unintended reactions, such as the peeling reaction, during reduction interfered in the oligosaccharide structural determination. Third, the structure of each oligosaccharide could be identified twice by using these two peaks in the chromatogram, providing a crosscheck for structural identification. Fourth, the retention times and MS^n^ mass spectra of two anomers belonging to one oligosaccharide isomer were not likely to be the same as those of two anomers belonging to another oligosaccharide isomer. The probability of the retention times and MS^n^ mass spectra of one isomer being the same as those of another isomer was considerably low, particularly after they were separated through multidimensional HPLC. These advantages enabled us to determine oligosaccharide isomer structures unambiguously.

LODES/MS^n^ involves the sequential collision induced dissociation (CID) of oligosaccharide sodium (or lithium) adducts in a mass spectrometer. The sequences of CID are guided by LODES which is derived from carbohydrate dissociation mechanisms^42–44^. The mechanisms of oligosaccharide sodium adducts used in this study are summarized as three propensity rules as follows.

1. Dehydration mainly occurs at the reducing end of oligosaccharides.
2. Cross-ring dissociation mainly occurs at the reducing end of oligosaccharides and follows the retro-aldol reaction. Fragmentation patterns from cross-ring dissociation can be used to determine the linkage position of the sugar at the reducing end. Details of fragmentation patterns are illustrated in Supplementary Information.
3. The cleavages of the glycosidic bond to produce B, C, Y and Z ions occur at any glycosidic bond (i.e., not limited to the reducing end). The notations B, C, Y, and Z were used according to the nomenclature of Domon and Costello^45^.

The dissociation mechanism of lithium adducts is similar to that of sodium adducts; however, the dehydration and cross-ring dissociation occurring at the nonreducing end was not neglectable. Thus, the O1 atoms of the monosaccharide at the reducing end labeled by ^18^O in some oligosaccharides are necessary when lithium adducts are used for structural determination.

**Figure 2:**
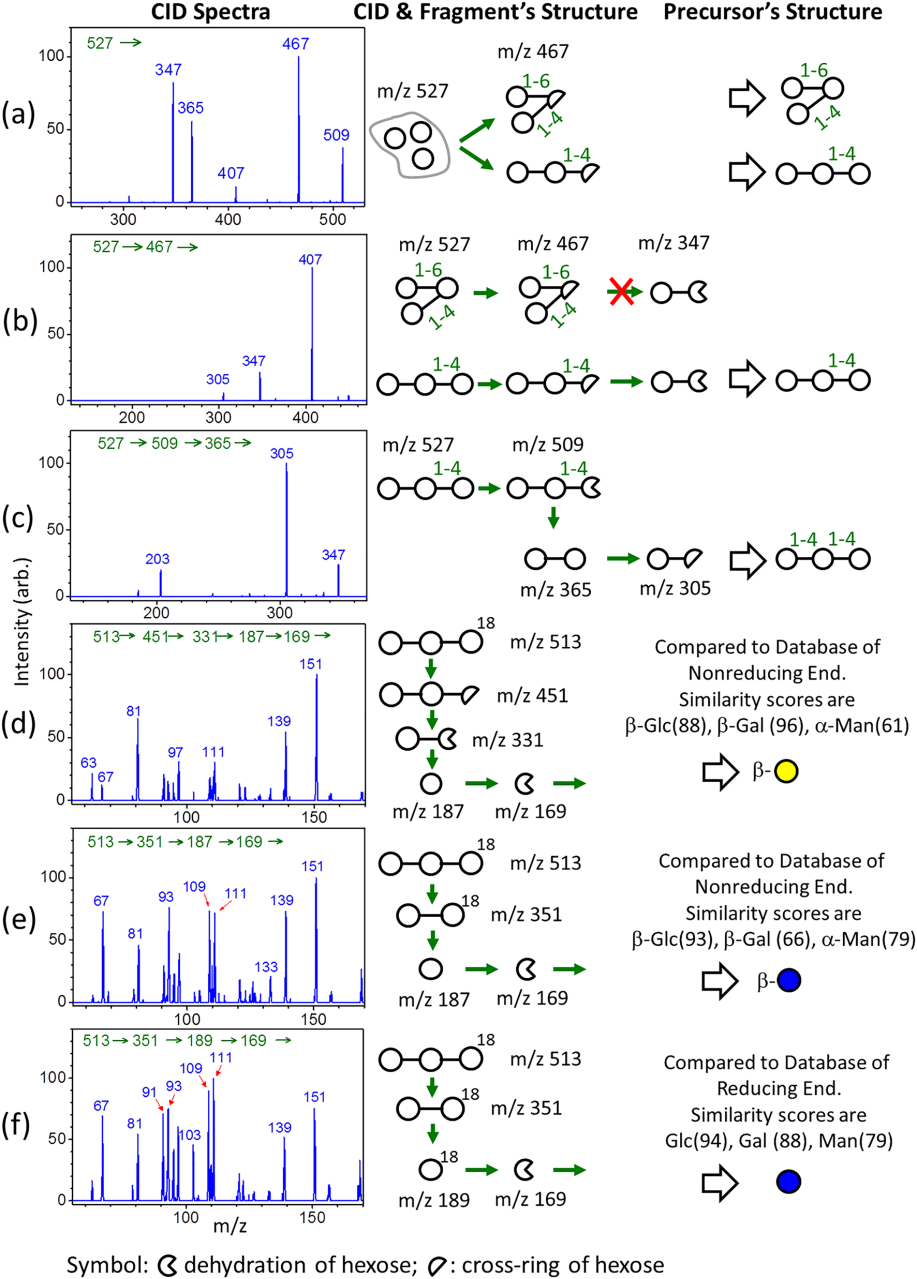
Structural determination of the peak at retention time 40.1 min shown in Fig. 1b by using LODES/MS^n^. CID spectra, CID sequence, and possible fragment and precursor structures are illustrated in the left, middle, and right columns of (**a–f**), respectively. Hexoses circulated by gray line in (**a**) represent the linkage between these hexoses are not determined. Green arrows represent CID.

In this study, we used the trisaccharide β-Gal-(1 → 4)-β-Glc-(1 → 4)-Glc as an example to illustrate how the structures of oligosaccharides are determined using LODES/MS^n^. The CID spectra of the oligosaccharide of the peak at the retention time 40.1 min in Fig. 1b are shown in Fig. 2. The mass spectrum, presented on the left side of Fig. 2a, shows the fragments produced from CID of the precursor ion *m/z* 527 [sodium adduct of (Hex)_3_]. The loss of neutral *m* = 60 from the precursor ion resulting in the fragment ion *m/z* 467 represented cross-ring dissociation at the reducing end (rule 2). The trisaccharide must be linear with a linkage of 1 → 4 at the reducing end or branched with 1 → 6 and 1 → 4 linkages at the reducing end, according to the retro-aldol reaction (details of fragmentation patterns are provided in Supplementary Information). The CID sequence and structures of fragments are illustrated in the middle of Fig. 2a; the possible precursor structures derived from these fragments are illustrated on the right side of Fig. 2a. Ion *m/z* 347 found in the CID sequence 527 → 467 → fragments (left side of Fig. 2b) indicated that the trisaccharide was linear because a branched trisaccharide with 1 → 6 and 1 → 4 linkages cannot produce the fragment ion *m/z* 347 from this CID sequence (middle of Fig. 2b). The CID sequence 527 → 509 → 365 represented dehydration at the reducing end (rule 1), followed by glycosidic bond cleavage (rule 3), as illustrated in the middle of Fig. 2c. Ion *m/z* 365 produced from this CID sequence was the disaccharide at the nonreducing end of the trisaccharide. The CID spectrum of this disaccharide, as illustrated on the left side of Fig. 2c, showed a high intensity of ion *m/z* 305 (the loss of neutral *m* = 60 from ion *m/z* 365), indicating that the linkage of the disaccharide is 1 → 4 according to fragmentation patterns obtained from the retro-aldol reaction (rule 2). These three CID spectra of sodium adducts (Fig. 2a–c) suggested that the trisaccharide is Hex-(1 → 4)-Hex-(1 → 4)-Hex. To determine the stereoisomer of each monosaccharide, the O1 atom of hexose at the reducing end of the trisaccharide was ^18^O labeled, and the CID spectra of ^18^O labelled trisaccharide lithium adducts were investigated. The hexose lithium adducts, with ion *m/z* 187 or 189, produced from the CID sequences 513 → 451 → 331 → 187, 513 → 351 → 187, and 513 → 351 → 189 represented the hexose at the nonreducing end, center, and reducing end of the trisaccharide, respectively. The CID spectra of these hexose monosaccharides are compared to the monosaccharide database provided in Supplementary Information. Spectrum similarities were calculated in the comparison. Method of spectrum similarity calculations for structural identification has been described in our previous report^37^. The similarity scores are shown in Fig. 2. The monosaccharide (glucose, galactose, or mannose) which has the highest similarity score is identified as the stereoisomer of the monosaccharide. The results show that the hexose at the nonreducing end, center, and reducing end of the trisaccharide were β-Gal, β-Glc, and Glc, respectively. Consequently, the entire trisaccharide was determined to be β-Gal-(1 → 4)-β-Glc-(1 → 4)-Glc. This trisaccharide, β-Gal-(1 → 4)-β-Glc-(1 → 4)-Glc, has not been found before, and its structure is unusual: it does not contain a lactose at the reducing end. In addition to the presence in bovine milk, we found that this trisaccharide was present in human milk and caprine milk as well, as illustrated in the chromatograms of Fig. 1a, c. The complete CID spectra of all the other oligosaccharides reported in this study are illustrated in Supplementary Information.

**Figure 3:**
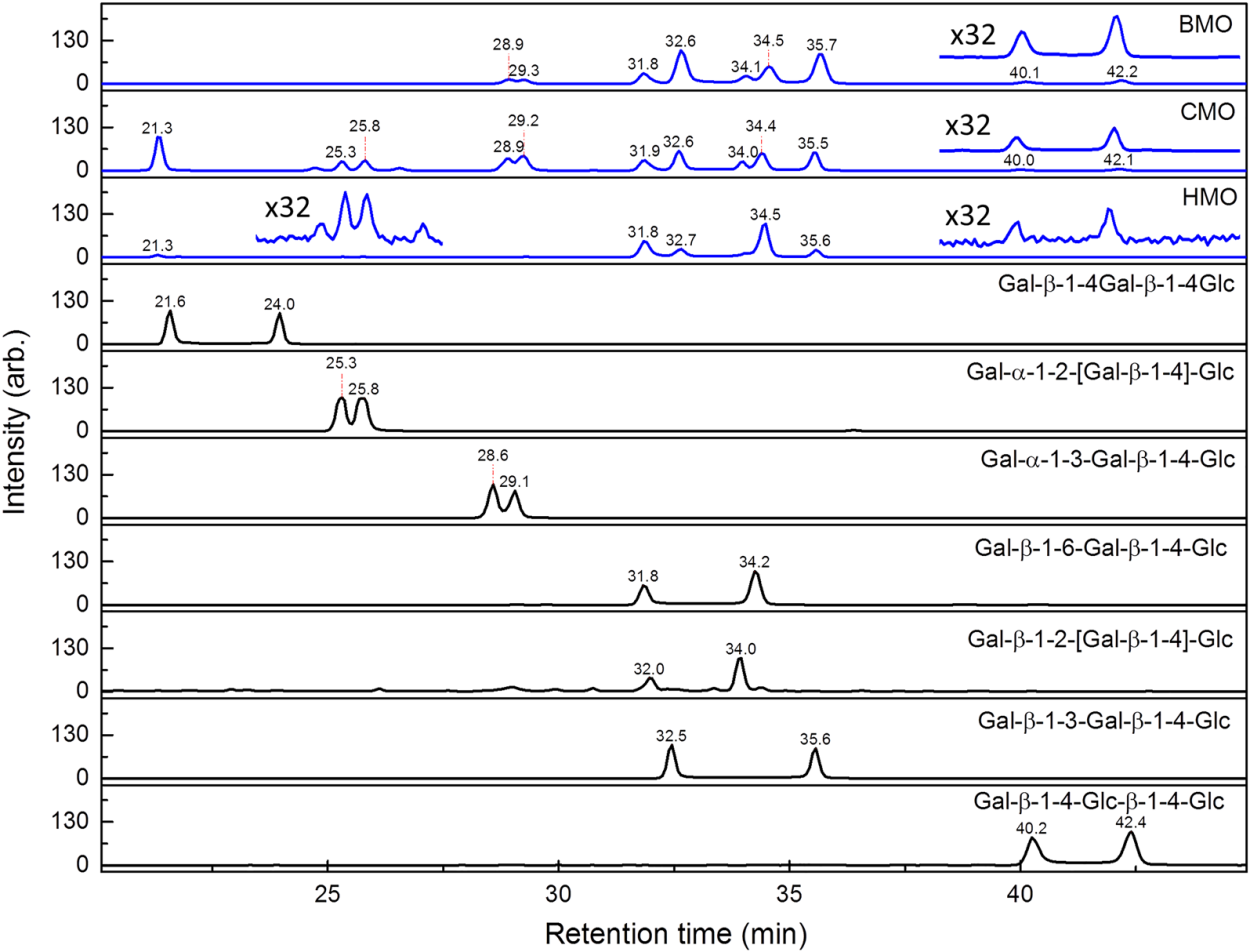
Chromatograms of ion *m/z* 527, sodium adducts of (Hex)_3_, of human milk oligosaccharides (HMO), bovine milk oligosaccharides (BMO), caprine milk oligosaccharides (CMO), and various chemically synthesized (Hex)_3_.

**Figure 4:**
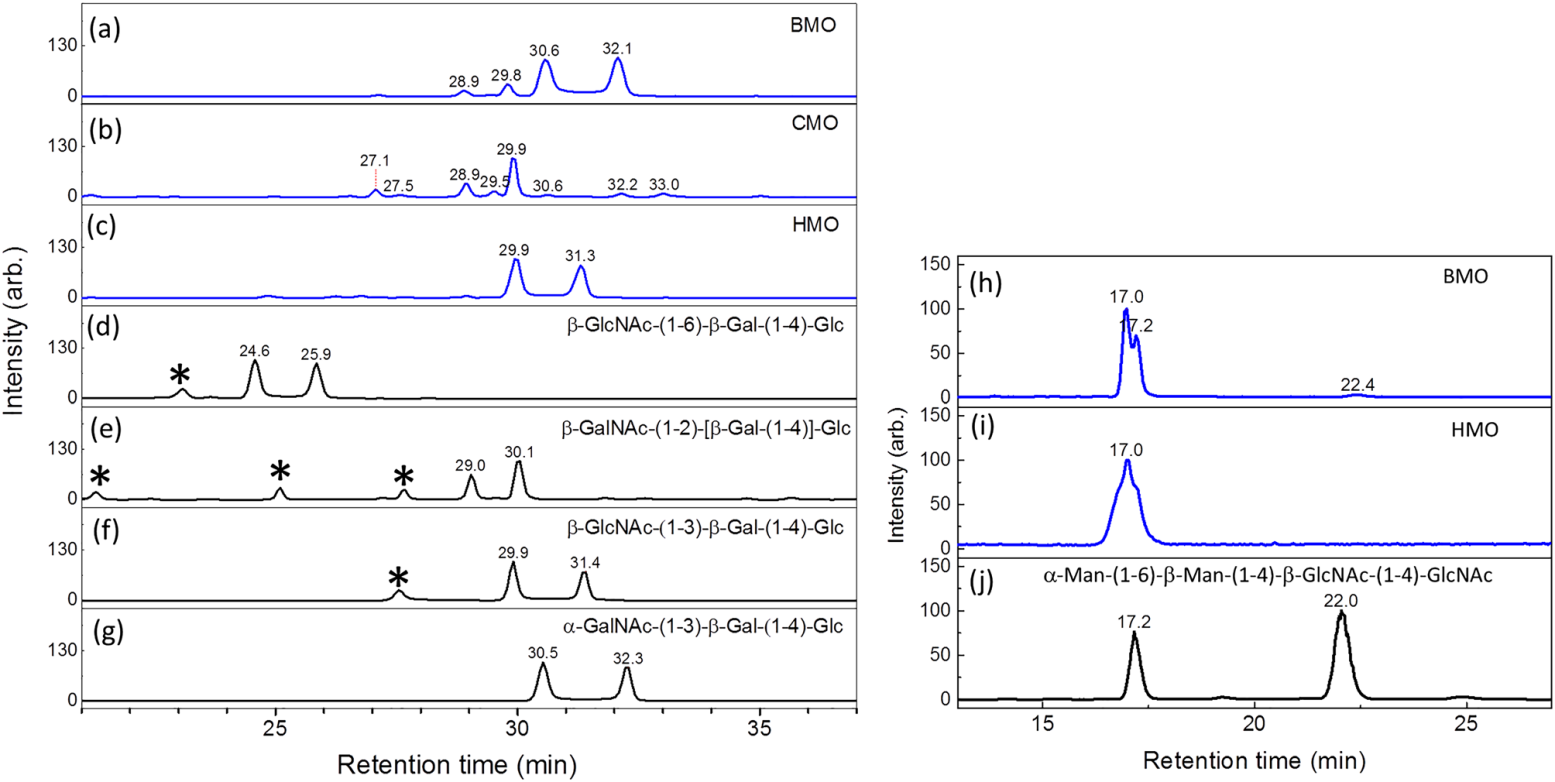
(**a–g**) Chromatograms of ion *m/z* 568, sodium adducts of (Hex)_2_HexNAc, of human milk oligosaccharides (HMO), bovine milk oligosaccharides (BMO), caprine milk oligosaccharides (CMO), and various chemically synthesized (Hex)_2_HexNAc. Peaks with symbol * represent impurity. (**h–j**): Chromatograms of ion *m/z* 771, sodium adducts of (Hex)_2_(HexNAc)_2_, of bovine milk oligosaccharides, human milk oligosaccharides (HMO), and synthesized α-Man-(1–6)-β-Man-(1–4)-β-GlcNAc-(1–4)-GlcNAc. α-Man-(1–6)-β-Man-(1–4)-β-GlcNAc-(1–4)-GlcNAc standard has two peaks (17.2 and 22 min), as shown in (**j**). In bovine milk, we can find a small peak at 22 min. The other peak of α-Man-(1–6)-β-Man-(1–4)-β-GlcNAc-(1–4)-GlcNAc (17.2 min) in bovine milk overlaps with another tetrasaccharide (retention time 17.0 and 17.2 min).

In addition to the structural determination using CID spectra, the structures were double checked using an orthogonal method: comparison to the retention time in chromatogram and CID mass spectra of synthesized trisaccharides. PGC column can separate most oligosaccharide isomers and generate reproducible chromatograms, it has been used to construct chromatogram database of various glycans^46–50^. The comparison for structural determination is based on the following three criteria. (1) There are two peaks (i.e., α and β anomers) for each isomer. The retention times of these two peaks in the chromatogram of selected *m/*z value must be within 0.5% of the retention times of the synthesized trisaccharide. (2) The CID MS^2^ mass spectra at these two retention times must be similar to that of the synthesized trisaccharide. (3) The relative intensity of these two peaks must be close to that of the synthesized trisaccharide. This is because α and β anomers change to each other and reach equilibrium through mutarotation. The relative abundance of these two anomers must be similar if the temperature and solvent are similar. Figures 3 and 4 show the chromatograms of trisaccharides and tetrasaccharides extracted from human, bovine and caprine milk and the chromatograms of synthesized trisaccharides. The CID MS^2^ spectra and the NMR spectra of the synthesized trisaccharides are illustrated in Supplementary Information. The comparison based on the aforementioned criteria supports the structural determination made by LODES/MS^n^.

**Figure 5:**
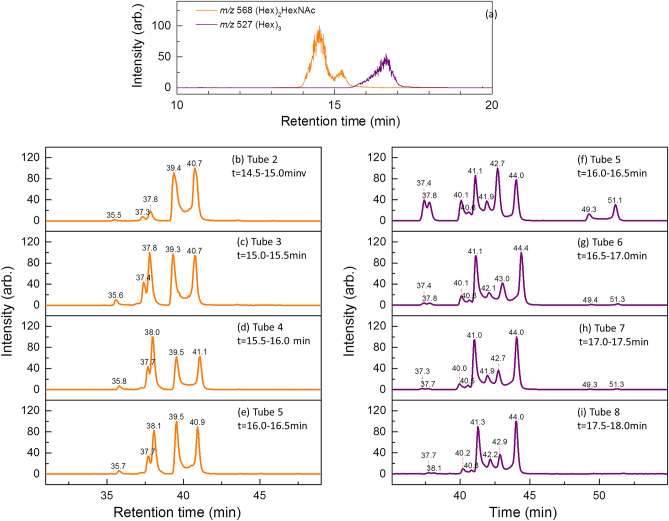
(**a**) Chromatograms of ions *m/z* 527, (Hex)_3_ and 568, (Hex)_3_HexNAc, separated by HPLC with amide-80 column of bovine milk. (**b–e**): Chromatograms of ion *m/z* 568. (**f–i**): Chromatograms of ion *m/z* 527. In (**b–i**), the isomers in each fraction collected from the eluents of amide-80 column were separated by HPLC with PGC column. The duration that each fraction collected from the eluents of amide-80 column, corresponding to retention time in (**a**), is illustrated for each tube. Note that the absolute retention times are different from that of Figs. 1–4 because of different HPLC apparatus and different lengths of tubing in HPLC were used.

In addition to the aforementioned orthogonal method, part of the structures assigned by LODES/MS^n^ can be further verified using the following chromatograms. Figure 5a shows the chromatograms of ions *m/z* 527 and 568 separated by HPLC with amide-80 column of bovine milk. There are many isomers of Hex_3_ and HexNAcHex_2_ covered by the curves of ions *m/z* 527 and 568, respectively, but these isomers were not separated well from each other. The eluents from amide-80 column were collected every 30 s. The collected fractions were concentrated and injected into HPCL with PGC column. The chromatograms of ions m/z 527 and 568 by using PGC column are illustrated in Fig. 5b–e and f–i, respectively. Although isomers are not separated well by amide-80 column, they are partially separated. Therefore, the isomer distribution in front part of the curve *m/z* 527 (or *m/z* 568) in Fig. 5a is different from the isomer distribution in rear part of the same curve. The relative abundances of these isomers change along the retention time in Fig. 5a, as illustrated by the change of relative isomers between different collected fractions (i.e., different tubes) in Fig. 5b–e and f–i. However, the relative abundances of α and β anomers of each isomer remain the same along the retention time (because α and β anomers change to each other and reach equilibrium through mutarotation). For example, the relative intensity of peaks at retention time t = 37.4 and 37.8 min in Fig. 5b–e remains the same, although absolute intensity are different in different collected fractions. Thus, we can assign peaks at t = 37.4 and 37.8 min belong to one isomers according to these relative intensities. Analogous analysis shows that peaks at t = 44.1 and 44.0, peaks at 40.1 and 42.7 min, peaks at 40.6 and 41.9 min and 49.3 and 51.1 min in Fig. 5f–i belong to four different isomers, respectively, and peaks at 39.4 and 40.7 min, and 37.3 and 37.8 min in Fig. 5b–e belong to two different isomers, respectively. The results are consistent to the structural analysis using LODES/MS^n^.

Five (Hex)_3_ isomers were found in human and bovine milk and six (Hex)_3_ isomers were discovered in caprine milk (Fig. 1a–c). We did not find the trisaccharide β-Gal-(1 → 4)-β-Gal-(1 → 4)-Glc in human, bovine, or caprine milk. The existence of this trisaccharide has been found previously in bovine milk^31,32,51,52^ and caprine milk^53^. Rudd et al. used enzyme digestion and mass spectrometry to identify the structure of β-Gal-(1 → 4)-β-Gal-(1 → 4)-Glc in bovine milk^31^; however, these authors did not report the discovery of this oligosaccharide in bovine milk in their later report^33^. We examined the presence of this trisaccharide by using chemically synthesized β-Gal-(1 → 4)-β-Gal-(1 → 4)-Glc. The retention times of the trisaccharide β-Gal-(1 → 4)-β-Gal-(1 → 4)-Glc in the chromatogram were located at *t* = 21.6 and 24.0 min (Fig. 3), but no signal was observed in the chromatograms of human, bovine, or caprine milk at the same retention time.

The trisaccharide β-GlcNAc-(1 → 3)-β-Gal-(1 → 4)-Glc, which is a crucial precursor in human milk oligosaccharides for subsequent synthesis, is the dominant species of (Hex)_2_HexNAc in human milk. We did not find this trisaccharide in bovine and caprine milk, as observed by a comparison of the Hex_2_HexNAc chromatograms of human, bovine, and caprine milk in Fig. 1d–f, although the existence of this trisaccharide in bovine milk was reported in previous studies^31,33,34,53^. The trisaccharide β-GlcNAc-(1 → 6)-β-Gal-(1 → 4)-Glc was not found in human, bovine, or caprine milk^31,33^. This trisaccharide was found in bovine milk in one previous study^32^, however, it was not reported to be present as an oligosaccharide in bovine milk by the same authors in their later study^34^. In recent study, this trisaccharide was found in caprine milk, but not in bovine milk^53^. We used chemically synthesized β-GlcNAc-(1 → 6)-β-Gal-(1 → 4)-Glc to double-check whether this trisaccharide was present. No signals at the same retention times of β-GlcNAc-(1 → 6)-β-Gal-(1 → 4)-Glc were observed in the chromatograms of human, bovine, or caprine milk (Fig. 3). Three other trisaccharides, namely α-GalNAc-(1 → 3)-β-Gal-(1 → 4)-Glc, β-GalNAc-(1 → 3)-β-Gal-(1 → 4)-Glc, and β-GalNAc-(1 → 2)-[β-Gal-(1 → 4)]-Glc, were identified in bovine milk in the present study. The presence of the first trisaccharide in bovine milk has been reported in many studies^25,31–34^, the second trisaccharide was only reported in one study^54^, and the third trisaccharide was not reported before. Trisaccharide β-GalNAc-(1 → 4)-β-Gal-(1 → 4)-Glc was reported in bovine and caprine milk in recent study^53^, but was not found in this study.

### Tetrasaccharides

**Figure 6:**
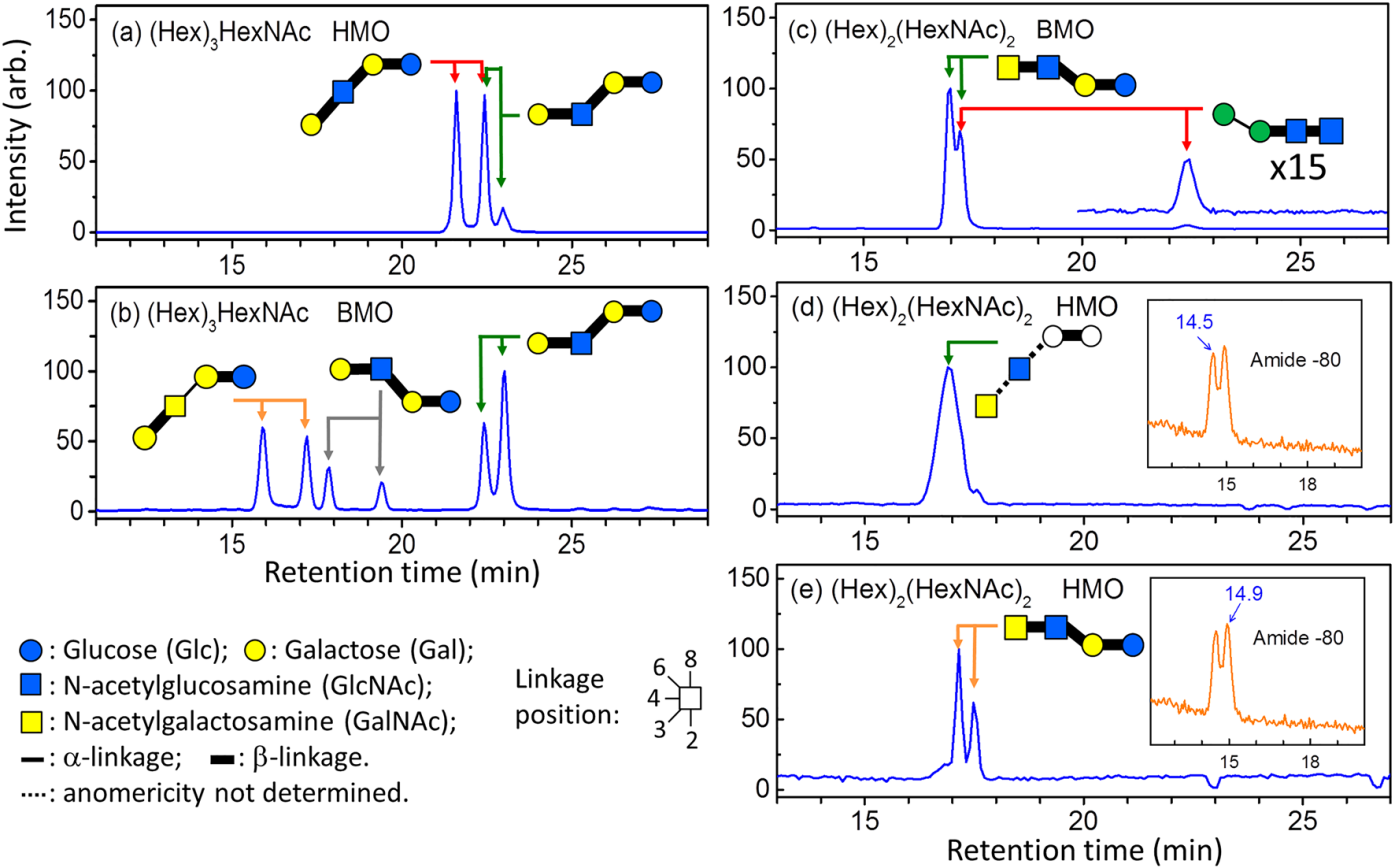
PGC chromatograms of tetrasaccharides (**a, b**) ion *m/z* 730 [sodium adducts of (Hex)_3_HexNAc]; (**c–e**) ion *m/z* 771 [sodium adducts of (Hex)_2_(HexNAc)_2_] for human milk oligosaccharides (HMO) and bovine milk oligosaccharides (BMO). The inserts in (**d, e**) are the amide-80 chromatograms of ion *m/z* 771, in which eluents at the retention time of 14.5 and 14.9 min were collected separately and injected into a PGC column to obtain the chromatograms shown in (**d**, **e**), respectively. The fraction collected from the amide-80 column was chosen such that all oligosaccharides found in this study are shown in a single chromatogram for compact display. The relative intensity in this chromatogram does not represent the relative abundance of these oligosaccharides in milk. The oligosaccharide structure for each peak in chromatograms was determined using LODES/MS^n^. Some of tetrasaccharide structures were crosschecked using the chromatogram retention time of synthesized oligosaccharides. Details of the structural determination using LODES/MS^n^ are described in Supplementary Information.

Figure 6 shows the chromatograms of tetrasaccharide ion *m/z* 730 [sodium adducts of (Hex)_3_HexNAc] and *m/z* 771 [sodium adducts of (Hex)_2_(HexNAc)_2_]. In addition to the tetrasaccharides β-Gal-(1 → 4)-β-GlcNAc-(1 → 3)-β-Gal-(1 → 4)-Glc, and β-Gal-(1 → 3)-β-GlcNAc-(1 → 3)-β-Gal-(1 → 4)-Glc, which have been found in human milk^18,19,22,24,55^, we found two unreported tetrasaccharides, namely β-GalNAc-(1 → 4)-β-GlcNAc-(1 → 6)-β-Gal-(1 → 4)-Glc and GalNAc-(1 → 3)-GlcNAc-(1 → 3)-β-Hex-(1 → 4)-Hex, in human milk in this study. Among the five tetrasaccharides we found in bovine milk, namely β-Gal-(1 → 4)-β-GlcNAc-(1 → 3)-β-Gal-(1 → 4)-Glc, β-Gal-(1 → 4)-β-GlcNAc-(1 → 6)-β-Gal-(1 → 4)-Glc, β-Gal-(1 → 3)-α-GalNAc-(1 → 3)-β-Gal-(1 → 4)-Glc, β-GalNAc-(1 → 4)-β-GlcNAc-(1 → 6)-β-Gal-(1 → 4)-Glc, and α-Man-(1 → 6)-β-Man-(1 → 4)-β-GlcNAc-(1 → 4)-GlcNAc, only the first one had been reported previously^31,33,34^. The CID spectra used for the structural identification of these tetrasaccharides by using LODES/MS^n^ and the crosschecking of part of these tetrasaccharide structures by using the chromatogram retention time of synthesized tetrasaccharides are shown in Supplementary Information. The tetrasaccharide β-Gal-(1 → 4)-β-GalNAc-(1 → 4)-β-Gal-(1 → 4)-Glc was found in bovine and caperine milk in recent study^53^. This tetrasaccharide is the elongation of the trisaccharide β-GalNAc-(1 → 4)-β-Gal-(1 → 4)-Glc. These trisaccharide and tetrasaccharide were not found in this study.

## Discussion

**Figure 7:**
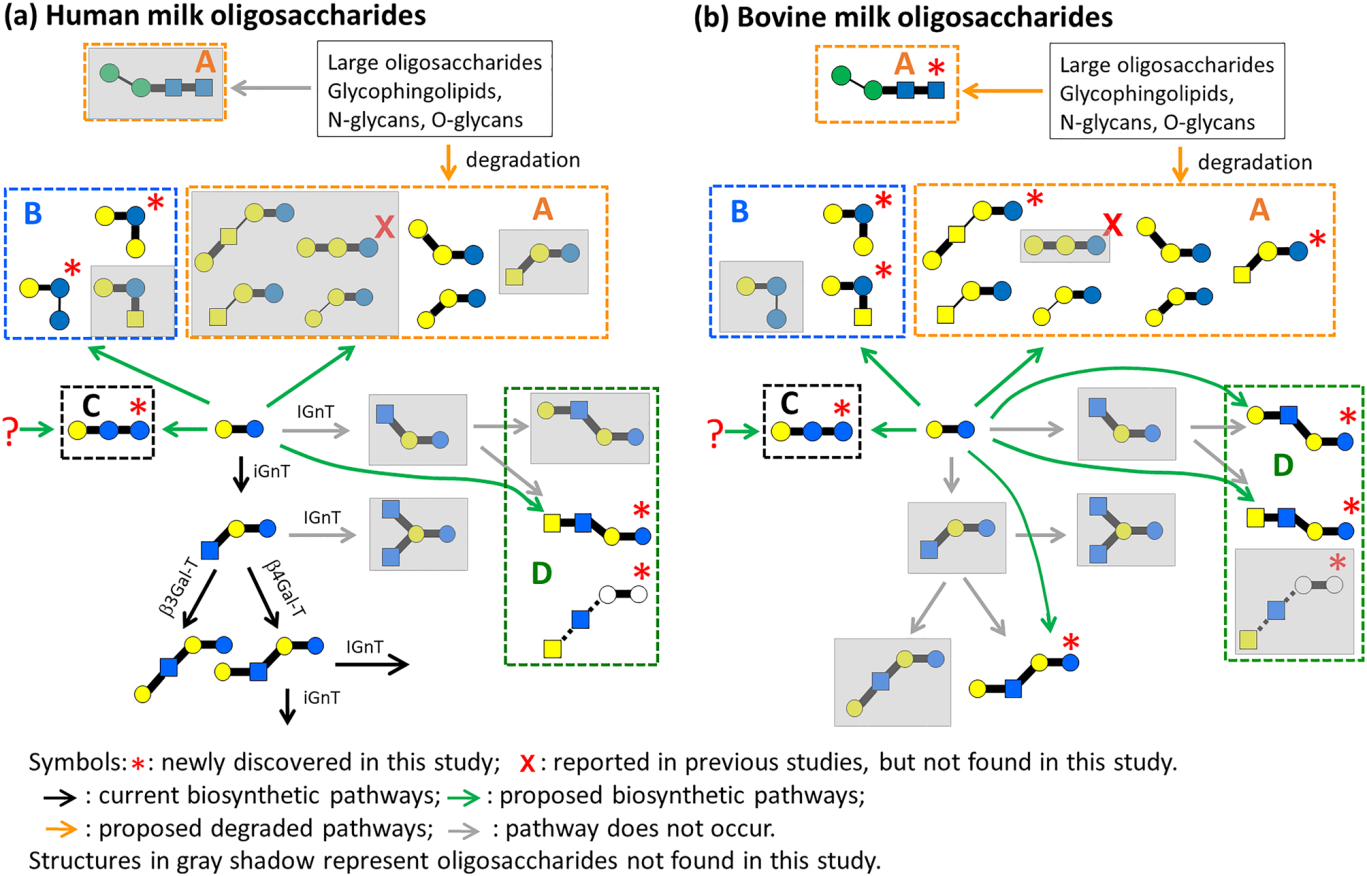
Biosynthetic pathways of neutral trisaccharides and tetrasaccharides consisting glucose, galactose, mannose, N-acetylglucosamine, and N-acetylgalactosamine. (**a**) Human milk oligosaccharides and (**b**) bovine milk oligosaccharides. To easily compare human and bovine milk oligosaccharides, all oligosaccharides discussed in this study are illustrated, and they are shown at the same positions in both biosynthetic pathways. Oligosaccharides in gray shadow were not found in this study. The discovered oligosaccharides are classified into four groups. Oligosaccharides in group A are generated by the elongation of lactose or the degradation from large oligosaccharides. Oligosaccharides in group B have lactrose at reducing end, but they have unusual linkages which are not found in other oligosaccharides. Oligosaccharide in group C does not have lactose at the reducing end. Oligosaccahrides in group D are tetrasaccharides, some of the trisaccahrides which lead to these tetrasaccharides from lactose were not found.

The current biosynthesis pathways of HMOs were proposed on the basis of the observation of lactose at the reducing end and linkages found in free oligosaccharides in human milk. The elongation of lactose to generate various free oligosaccharides in milk was made through the synthesis by the following four enzymes: iGnT (β-GlcNAc-(1 → 3)-Gal extension), IGnT (β-GlcNAc-(1 → 6)-Gal branching), β3Gal-T (β-Gal-(1 → 3)-GlcNAc type 1 termination), and β4Gal-T (β-Gal-(1 → 4)-GlcNAc type 2 formation)^2,6,9,56^, as illustrated in Fig. 7a. Among these four enzyme, glycotransferase IGnT (β-GlcNAc-(1 → 6)-Gal branching) only transfers GlcNAc onto Gal after the completion of β-GlcNAc-(1 → 3)-Gal extension by glycotransferase iGnT. Most free oligosaccharides in human milk reported in previous studies can be synthesized in a systematic manner by using these four enzymes, except the two reported trisaccharides, namely β-Gal-(1 → 3)-β-Gal-(1 → 4)-Glc and β-Gal-(1 → 6)-β-Gal-(1 → 4)-Glc. Recently, a new class of human milk oligosaccharides which branch at the terminal galactose of 6'-galactosyllactose and extended by oligolactose was reported^57^. This new types of oligosaccharides cannot be explained by the aforementioned biosynthesis. The number of free oligosaccharides, the structures of which have been identified in previous studies, is considerably lower in bovine or caprine milk than in human milk. Nevertheless, most of the free oligosaccharides in bovine and caprine milk reported in previous studies had lactose at the reducing end.

Although most oligosaccharides in milk have lactose at the reducing end, milk oligosaccharides lacking lactose at the reducing end have been reported. A trisaccharide was found in α-Neu5Ac-(2 → 6)-β-Gal-(1 → 4)-GlcNAc in caprine colostrum^58^; two disaccharides, β-Gal-(1 → 4)-GlcNAc and β-GalNAc-(1 → 4)-Glc, in bovine colostrum were identified^59^; a sialyl oligosaccharide phosphate, α-NeuAc-(2 → 6)-β-Gal-(1 → 4)-GlcNAc-P, was found in bovine colostrum^60^; and a tetrasaccharide, α-NeuAc-(2 → 3)-β-Gal-(1 → 3)-[α-Fuc-(1 → 4)]-GlcNAc, and a pentasaccharide α-NeuAc-(2 → 3)-β-Gal-(1 → 3)-[α-Fuc-(1 → 4)]-β-GlcNAc-(1 → 3)-Gal were found in human milk^61^. The discovery of these oligosaccharides raises the question as to how these oligosaccharides without a lactose moiety at the reducing end are synthesized. Kitagawa et al. suggested two possibilities^61^. One possibility is that they are synthesized through the sequential transfer of various monosaccharides to N-acetylglucosamine or galactose, catalyzed by the aforementioned glycosyltransferases. Notably, except disaccharides β-GalNAc-(1 → 4)-Glc, all these oligosaccharides lacking lactose at the reducing end have linkages related to the aforementioned four enzymes. The other possibility is that the oligosaccharides lacking lactose are the degradation products of large free oligosaccharides by respective endoglycosidases.

The synthesis of several oligosaccharides discovered in this study cannot be explained by the two possibilities proposed by Kitagawa et al. Trisaccharides and tetrasaccharides in human milk found in this study and the corresponding biosynthetic pathways are illustrated in Fig. 7a. Oligosaccharides that follow the current biosynthetic pathway are illustrated at the end of black arrows in Fig. 7a. Other oligosaccharides that cannot be produced by the current biosynthetic pathways, as illustrated at the end of green and orange arrows in Fig. 7a, can be classified into four groups. Oligosaccharides in group A (circulated by orange dash line), which have been found in previous studies, have lactose at the reducing end. The oligosaccharides in group A can be presented as moieties in other large glycans (e.g., N-glycans, O-glycans, and glycosphingolipids). They can be produced either by the elongation of lactose by undiscovered enzymes or the degradation from large glycans by other undiscovered enzymes. Oligosaccharides in group B (circulated by blue dash line) were identified in this study. Analogous to oligosaccharides in group A, oligosaccharides in group B have lactose at the reducing end. However, the linkages of the extension from lactose is unusual: oligosaccharides in group B have a branch with the linkage of β-Gal-(1 → 2)-Glc or α-Glc-(1 → 2)-Glc. These types of linkages are not found in any large oligosaccharides in milk, N-glycans, O-glycans, or glycosphingolipids, indicating they are not the degradation products of large oligosaccharides. A trisaccharide, β-Gal-(1 → 4)-Glc-β-Glc-(1 → 4)-Glc, found in this study is particular unusual and was classified into group C (circulated by black dash line). This trisaccharide does not have lactose at the reducing end, and it is not the moiety of any large oligosaccharides. This trisaccharide could be an extension of glucose to lactose at the reducing end or it might not be related to the extension of lactose (e.g., it starts from the extension at the nonreducing end of cellobiose instead of lactose). Group D consists of two tetrasaccharides. The tetrasaccharide GalNAc-(1 → 3)-GlcNAc-(1 → 3)-Hex-β-(1 → 4)-Hex could be the elongation of the trisaccharide β-GlcNAc-(1 → 3)-Gal-β-(1 → 4)-Glc by an enzyme that does not belong to the aforementioned four enzymes. The other tetrasaccharide is β-GalNAc-(1 → 4)-β-GlcNAc-(1 → 6)-Gal-β-(1 → 4)-Glc. However, the trisaccharide β-GlcNAc-(1 → 6)-Gal-β-(1 → 4)-Glc was not found. These findings suggest two possibilities. One possibility is that GalNAc glycosyltransferase is highly active such that no substrate (trisaccharide β-GlcNAc-(1 → 6)-Gal-β-(1 → 4)-Glc) remains. The other possibility is that tetrasaccharide β-GalNAc-(1 → 4)-β-GlcNAc-(1 → 6)-Gal-β-(1 → 4)-Glc was not synthesized through trisaccharide β-GlcNAc-(1 → 6)-Gal-β-(1 → 4)-Glc. Instead of the addition of only one monosaccharide in each step, transfer of the disaccharide β-GalNAc-(1 → 4)-GlcNAc onto lactose occurs.

Trisaccharides and tetrasaccharides in bovine milk and the corresponding biosynthetic pathways are shown in Fig. 7b. The biosynthetic pathways of oligosaccharides found in human milk considerably differ from those of free oligosaccharides in bovine milk. Figure 7b shows that the trisaccharide β-GlcNAc-(1 → 3)-Gal-β-(1 → 4)-Glc, which is an important precursor trisaccharide for subsequent synthesis in human milk free oligosaccharides, was not found in bovine milk. Many tetrasaccharides found in bovine milk are not synthesized through trisaccharides. Instead of the step-by-step addition of monosaccharides, they are likely to be synthesized through the addition of disaccharides onto the nonreducing end of lactose. Many “unusual” free oligosaccharides in human milk were found in bovine and caprine milk. In particular, oligosaccharides in group A of bovine and caprine milk have more varieties than do those in human milk. The observation of these unusual oligosaccharides suggests that many undiscovered glycosyltransferases and glycosidases are involved in biosynthetic pathways. The structures of these oligosaccharides found in this study provide crucial clues relevant to the search for undiscovered enzymes and the modification of current biosynthetic pathways.

## Methods

The method used to extract free oligosaccharides from milk was similar to that described in our previous study^37^. Bovine milk was purchased from Experimental Farm, College of Biosources and Agriculture, National Taiwan University, Taiwan. Caprine milk was purchased from a local market, and human milk was obtained from donors. Folch solution and ethanol were used to remove fat and proteins in milk, respectively. First, Folch solution was added to milk in a centrifuge tube and mixed using a vortex mixer (VTX-3000, Mixer Uzusio, Tokyo, Japan) for 1 min. The mixture was centrifuged for 30 min at 4000 × *g* and 4 °C (High-speed Micro Refrigerated Centrifuge, CF15RN, Hitachi, Japan). Subsequently, the top layer of the aqueous solution containing oligosaccharides was collected, and ethanol (− 20 °C) was added to the collected aqueous solution. The solution of ethanol/aqueous mixture was maintained at − 20 °C overnight. The mixture was centrifuged for 30 min at 4000 × *g* and 4 °C, and the top oligosaccharide-rich layer was collected and dried in vacuum. Subsequently, the samples were further purified using two solid phase extractions (SPEs). The first SPE involved using C18 cartridges (C18 SPE column, 2000 mg/12 mL, S*Pure Pte. Ltd, Singapore) to remove remaining lipids and proteins, and the second SPE involved using PGC (porous graphitized carbon, 1000 mg/15 mL, S*Pure Pte, Ltd, Singapore) cartridges to remove lactose. In the second SPE, oligosaccharides without sialic acid were collected by eluting the cartridge by using one column volume of 20% ACN in deionized (DI) water. The collected sample was dried in vacuum and then dissolved in DI water for subsequent separation.

After SPE, oligosaccharides were size-selected through size exclusion chromatography (TOYOPEARL HW-40F, Tosoh Bioscience GmbH, Griesheim, Germany) followed by separation using an HPLC system (Dionex Ultimate 3000, Thermo Fisher Scientific, Waltham, MA USA) with a TSKgel amide-80 column (150 mm × 2.0 mm, particle size of 5 µm; Tosoh Bioscience GmbH, Griesheim, Germany). Finally, each fraction collected from the amide-80 column was separately injected into another HPLC with a PGC Hypercarb column (2.1 mm × 100 mm, particle size of 3 µm; Thermo Fisher Scientific, Waltham, MA, USA) for further separation. For trisaccharides, methanol was added to the eluents from the PGC column, and the mixture was sent into a linear ion trap mass spectrometer (LTQ XL, Thermo Fisher Scientific, Waltham, MA USA) directly for online structural determination. For tetrasaccharides, eluents from the PGC column were sent into a fraction collector (FC204, Gilson, Middleton, WI, USA). Fractions collected from the fraction collector were vacuum dried. Subsequently, the sample was dissolved in a 50:50 (vol/vol) water/methanol mixture and sent into a nanoelectrospray mass spectrometer for structural determination. Detailed settings of the mass spectrometer are described in Supplementary Information.

The method used to prepare ^18^O-labeled oligosaccharide has been described in previous studies^62,63^. For preparing ^18^O-labeled oligosaccharides, 500 µL of the 3 mL sample was vacuum dried, and then 0.4 µL acetyl chloride and 50 µL H_2_^18^O were added to the dried sample. This solution was kept in a sealed vial. The sealed vial was sat in a vacuum desiccator with silica gel for more than 3 weeks.

Collection of human milk form donors and the study of free oligosaccharides in human milk were approved by IRB on Biomedical Science Research, Academia Sinica, Taiwan (AS-IRB-19038V.2 2020-06-17). All experiments were performed in accordance with relevant guidelines and regulations, and informed consents were obtained from all donors.

## Supplementary Information

Supplementary Information.

## Data Availability

The data supporting the findings of this study are available within the article and its Supplementary Information.
