# Supplementary material for: Unusual free oligosaccharides in human bovine and caprine milk
Source: Sci Rep. 2022 Jun 24;12:10790. doi: 10.1038/s41598-022-15140-7 (PMC9232581; doi:10.1038/s41598-022-15140-7)
Supplement: Supplementary file 1 — Supplementary Information. [file 41598_2022_15140_MOESM1_ESM.pdf]

Supplementary Information

Unusual free oligosaccharides in human bovine and caprine milk

Wei-Chien Weng[a,b], Hung-En Liao[a,c], Shih-Pei Huang[a], Shang-Ting Tsai[a], Hsu-Chen Hsu[a], Chia Yen Liew[a,d], Veeranjaneyulu Gannedi[e], Shang-Cheng Hung[e], Chi-Kung Ni*[a,b]

[a] Institute of Atomic and Molecular Sciences, Academia Sinica, Taipei, 10617, Taiwan

[b] Molecular Science and Technology, International Graduate Program, Academia Sinica and National Tsing Hua University, Hsinchu, 30013, Taiwan

[c] Department of Applied Chemistry, National Yang Ming Chiao Tung University, Hsinchu, 300, Taiwan

[d] International Graduate Program of Molecular Science and Technology, National Taiwan University and Taiwan International Graduate Program of Molecular Science and Technology, Academia Sinica, Taipei, 10617, Taiwan

[e] Genomic Research Center, Academia Sinica, Taipei, 11529, Taiwan

*Corresponding authors, e-mail address: ckni@po.iams.sinica.edu.tw

1. Experimental methods

(a) Materials

Disaccharides,α-Gal-(1→3)-Gal, β-Gal-(1→3)-Gal , β-Gal-(1→6)-Gal, α-GalNAc-(1→3)-Gal, β-GalNAc-(1→3)-Gal, β-GlcNAc-(1→6)-Gal, β-Gal-(1→3)-β-Gal-(1→4)-Glc, β-Gal-(1→4)-β-Gal-(1→4)-Glc, β-Gal-(1→6)-β-Gal-(1→4)-Glc, and α-GalNAc-(1→3)-β-Gal-(1→4)-Glc were purchased from Carbosynth Ltd (Compton, UK); β-GlcNAc-(1→3)-Gal were purchased from Synthose Inc. (Ontario, Canada); β-Gal-(1→2)-Glc, β-Gal-(1→3)-GalNAc, β-GalNAc-(1→2)-Glc, and β-GalNAc-(1→4)-GlcNAc were purchased from Omicron Biochemicals, Inc. (South Bend, In, USA); β-Gal-(1→3)-GlcNAc, β-Gal-(1→4)-GlcNAc, α-Gal-(1→3)-β-Gal-(1→4)-Glc, and β-GlcNAc-(1→3)-β-Gal-(1→4)-Glc were purchased from Elicity (Crolles, France); β-Gal-(1→4)-Glc was purchased from Sigma Aldrich (Merck KGaA, Munich, Germany); GalNAcb1-3GlcNAc was purchased from Peptide Institute Inc. (Osaka, Japan). The trisaccharides β-Gal-(1→2)-[β-Gal-(1→4)]-Glc, β-Gal-(1→4)-β-Glc-(1→4)-Glc, α-Glc-(1→2)-[β-Gal-(1→4)]-Glc, β-GalNAc-(1→2)-[β-Gal-(1→4)]-Glc, and β-GlcNAc-(1→6)-β-Gal-(1→4)-Glc were synthesized in our laboratory. The NMR spectra of the trisaccharides synthesized in our laboratory are presented in Supplementary Information, section 5.

(b) Extraction of free oligosaccharides from milk

The method to extract free oligosaccharides from milk was similar to that described to our previous studies[37]. The 20 mL of Folch solution (chloroform/methanol 2:1, v/v) were added to the 5 mL milk in a centrifuge tube, mixed by a vortex mixer (VTX-3000, Mixer Uzusio, Tokyo, Japan) for one minute. The mixture was centrifuged for 30 min at 4,000×g and 4 ℃ (High-speed Micro Refrigerated Centrifuge, CF15RN, Hitachi, Japan). The top layer aqueous solution containing oligosaccharides was collected, and ethanol with two volume of aqueous solution at -20℃ was added to the collected solution. The solution was kept at -20℃ overnight to precipitate the proteins, and then centrifuged for 30 min at 4,000×g and 4℃ to remove the precipitant. A rotary vaporator was used to remove the ethanol in oligosaccharide-rich solution. During the ethanol removing process, DI water was added to the solution when the volume of the solution was reduced to 1/3 of its original volume. The process was repeated twice. In some samples, the removal of fat by using Folch solution was replaced by ultrahigh speed centrifugation. No difference of oligosaccharides was found between these two methods.

The samples were further purified via solid phase extraction (SPE). SPE

involved C18 cartridges as the first step to remove the remaining lipids and proteins, PGC (porous graphitized carbon) cartridges as the second step to remove lactose. The C18 cartridges (C18 SPE column, 2000 mg/12 mL, S*Pure Pte. Ltd, Singapore) were first conditioned with one column volume of 100% ACN, followed by two column volume of DI water. Then previously prepared sample was added to a C18 cartridge, and the cartridge was washed by one column volume of DI water. The solution from C18 cartridges was collected for second step SPE. In the second step of SPE, PGC cartridges (PGC SPE column, 1000 mg/15 mL, S*Pure Pte, Ltd, Singapore) were first conditioned with one column volume of 80% ACN with 0.05% TFA in DI water, and then by two column volume of DI water before the solution collected from one C18 cartridge was added. Lactose was washed away by adding two column volume of DI water and one column volume of 5% ACN in DI water. The oligosaccharides without sialic acid were collected by eluting the cartridge using one column volume of 20% ACN in DI water; oligosaccharides containing sialic acid were collected separately by eluting the cartridge using one column volume of 40% ACN in DI water with 0.05% TFA. Only the sample eluted by using one column volume of 20% ACN in DI water was used in this study. The sample was then vacuum dried for the following HPLC separation.

In order to improve detection of the less abundant components as well as increase the possibility that each peak in chromatogram of HPLC contains only one oligosaccharide, multi-dimension chromatography was carried out. First, sample was size selected using a 1.75 m size exclusion column (TOYOPEARL HW-40F, Tosoh Bioscience GmbH, Griesheim, Germany). Eluent was collected every 30 minutes. The fractions collected from size exclusion were concentrated by vacuum centrifugal concentrator, and then pre-fractionated by chromatography using a TSKgel amide-80 column (150 mm × 2.0 mm, particle size of 5 µm, Tosoh Bioscience GmbH, Griesheim, Germany). Samples were eluted with a set HPLC system (Dionex Ultimate 3000, Thermo Fisher Scientific, Waltham, MA USA) with deionized water (A) and 100%ACN (B) with a flow rate of 0.2 mL/min and a gradient of 0.0 to 30.0 min, 75 to 50% B; 30.0–35.0 min, 50% B; 35.0–50.0 min, 75%B. The solution eluted from HPLC was collected in a test tube for every 30s. The process was repeated several times to collect enough samples. The solution in each test tube was concentrated by vacuum centrifugal concentrator.

For trisaccharides, sample collected from the pre-fractionation of amide-80 column was injected into the next stage HPLC with a porous graphitic carbon (PGC) Hypercarb column (2.1 mm × 100 mm, particle size of 3 µm, Thermo Fisher Scientific, Waltham, MA, USA) and mass spectrometer ((Dionex Ultimate 3000 and

LTQ XL, Thermo Fisher Scientific, Waltham, MA, USA) for online structural determination. Some isomers are difficult to be separated from each other by using PGC column, repeating separation using PGC column was performed. For example, the one anomer of β-Gal-(1→6)-β-Gal-(1→4)-Glc has retention time at 31.8 min with lower intensity, and the other anomer has retention time at 34.5 min with higher intensity. ON the other hand, one anomer of β-Gal-(1→4)-[β-Gal-(1→2)]-Glc has retention time at 31.8 min with higher intensity and it overlaps with one of the anomer of β-Gal-(1→6)-β-Gal-(1→4)-Glc. The other anomer has retention time around 34.1 min with lower intensity and it is very close to the retention time of the other anomer of β-Gal-(1→6)-β-Gal-(1→4)-Glc. To separate these two isomers, the fraction collected at retention time around 31.8 min and around 34.1-34.5 min were repeating injected into PGC column separately. Eventually, the eluent collected at retention time 31.8 min only contains β-Gal-(1→4)-[β-Gal-(1→2)]-Glc, and the eluent collected at retention time 34.5 min only contains β-Gal-(1→6)-β-Gal-(1→4)-Glc. These collected fractions are then injected into PGC column for online structural determination.

For tetrasaccharides, eluents from PGC was sent into a fraction collector (FC204, Gilson, Middleton, WI, USA). The fractions collected from fraction collector were vacuum dried. Then the sample was dissolved in a 50:50 (vol/vol) water/methanol mixture and sent into nanoelectrospray mass spectrometer for structural determination.

(c) HPLC-mass spectrometry

For trisaccharides, sample collected from amide-80 column was injected into the next stage HPLC-MS for the online structural determination. Liquid chromatography separation of oligosaccharides was achieved using a Hypercarb column at 25℃. The mobile phase for separating trisaccharides comprised (A) 0.1% (v/v%) aqueous formic acid and (B) HPLC-grade acetonitrile. The gradient of mobile phase changed linearly as follows: 0.0 to 5.0 min, 0 to 3% B; 5.0–60.0 min, 3 to 18% B; 60.0–65.0 min, 90%B; 65.0–80.0 min, 0%B. The injection volume of the sample was 10μL, and the mobile phase flow rate was 0.15 mL/min. The column eluate was mixed with 10^{-4} M NaCl or LiCl with flow rate 0.1 mL/min before infused into the ESI source. The MS conditions were optimized using the built-in semiautomatic tuning procedure in the Xcalibur software. The ESI source was operated at a temperature of 100℃ with 30 units of sheath gas flow, 0 units of auxiliary gas flow and 0 units sweep gas flow. The ion spray voltage was 5 kV, and the transfer capillary temperature was 275℃. The capillary voltage was 138 V, and the tube lens voltage was 249 V. The entire HPLC and mass spectrometer system is controlled by using Dinoex Chromatography

MS Link 2.14, Chromeleon Version 6.80 SR13, LTQ Tune Plus Version 2.7.0.1103 SP1, and Thermo Xcalibur 2.2 SP1.48 software from Thermo Fisher Scientific. No customization of these instruments was made.

Helium (He) gas was used as the buffer gas for the ion trap and the collision gas in CID. The pressure of He gas at the output of regulator connected to gas cylinder was set at the specification (40 psi). The pressure measured by the ion gauge in the vacuum chamber of mass spectrometer was 0.9×10^{-5} Torr. The MS^n experiments were performed using resonance excitation at an activation Q value of 0.25, an activation time of 30 ms, normalized collision energy 40%, and scan rate 16700/sec. The number of ions was regulated by injection time (50 ms) or automatic gain control (1×10^5 for all full scan, and 1×10^4 for MS^n). The precursor ion isolation width was set to 1 u. The CID spectra obtained in the MS^n experiments were used to determine the structures of oligosaccharides directly from HPLC.

(d) Nanoelectrospray-Mass spectrometry

For tetrasaccharides, eluents from PGC was sent into a fraction collector. The fractions collected from fraction collector were vacuum dried. A small part of each fraction collected from PGC column was dissolved in DI water and injected into PGC again to make sure each fraction contains only one isomer before sending into nanoelectrospray-mass spectrometry for structural identification. The rest part of each fraction was dissolved in a 50:50 (vol/vol) water/methanol mixture and sent into nanoelectrospray mass spectrometer for structural determination. A nanoelectrospray ionization instrument coupled to a linear ion trap mass spectrometer (LTQ XL, Thermo Fisher Scientific) was used for mass spectrometry. Specifically, 2 μL (the minimum volume for the emitter of ESI) of each sample was loaded into a borosilicate glass ESI emitter, which was produced in our laboratory using a P-97 (Sutter Instruments, Novato, CA, USA) Flaming/Brown micropipette puller. The ESI source voltage was 1.5 kV. In the mass spectrometer, the capillary voltage was 130 V, heated capillary temperature was 120℃, and tube-lens voltage was 230 V. Helium gas was used as a buffer gas for the ion trap as well as a collision gas in CID. The pressure of He gas at the output of regulator connected to gas cylinder was set at the specification (40 psi). The pressure measured by the ion gauge in the vacuum chamber of mass spectrometer was 0.9×10^{-5} Torr. The MS^n experiments were performed at an activation Q value of 0.25, an activation time of 30 ms, normalised collision energy 35-40%. The number of ions was regulated by injection time (10-20 ms) or automatic gain control (1×10^5 for full scan, and 1×10^4 for MS^n). The precursor ion isolation width was set to 1u.

2. Retro-aldol reactions and databases of monosaccharides and disaccharides

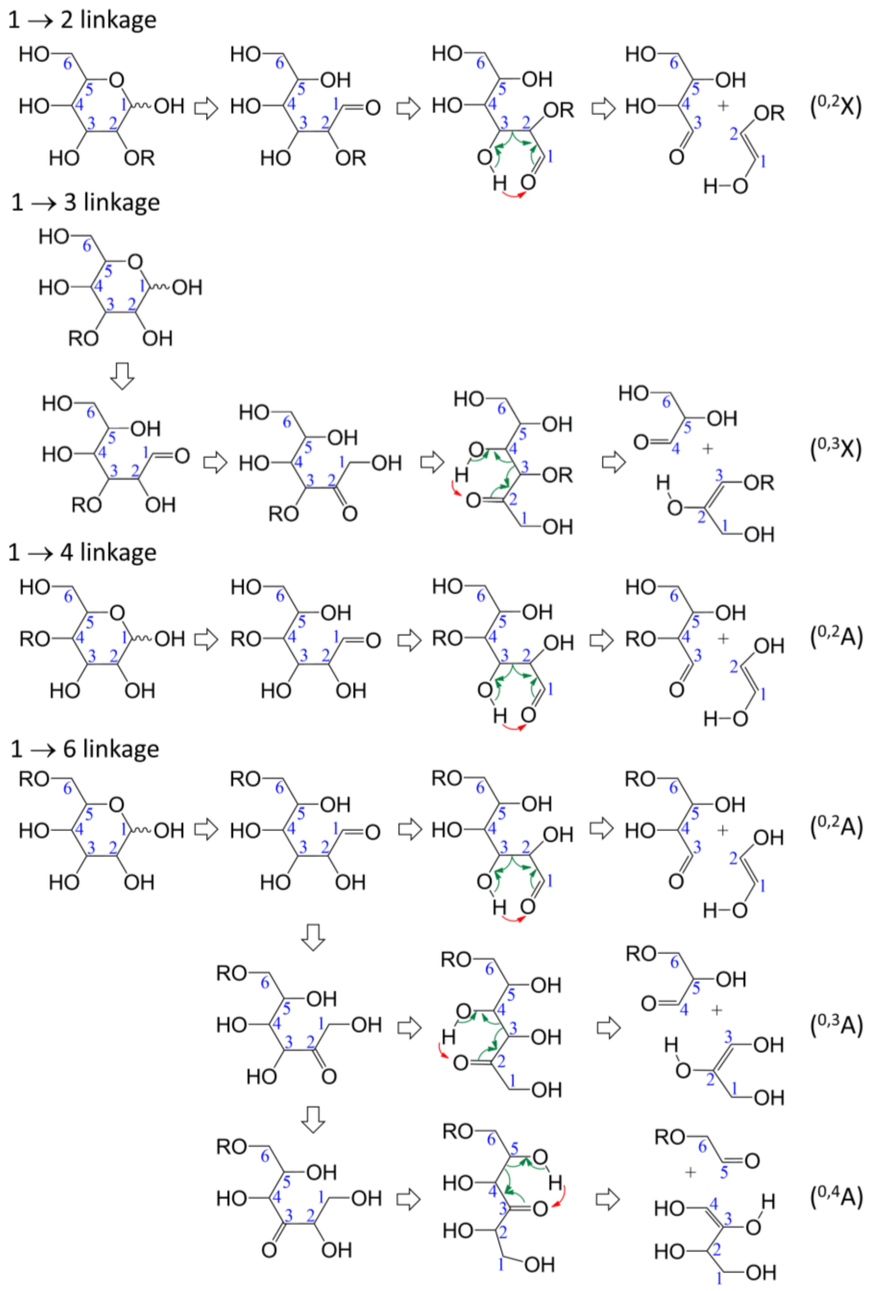

Fig. S2.1 Retro-aldol reaction of the hexose at the reducing end and connects to the other sugars through one glycosidic bond.

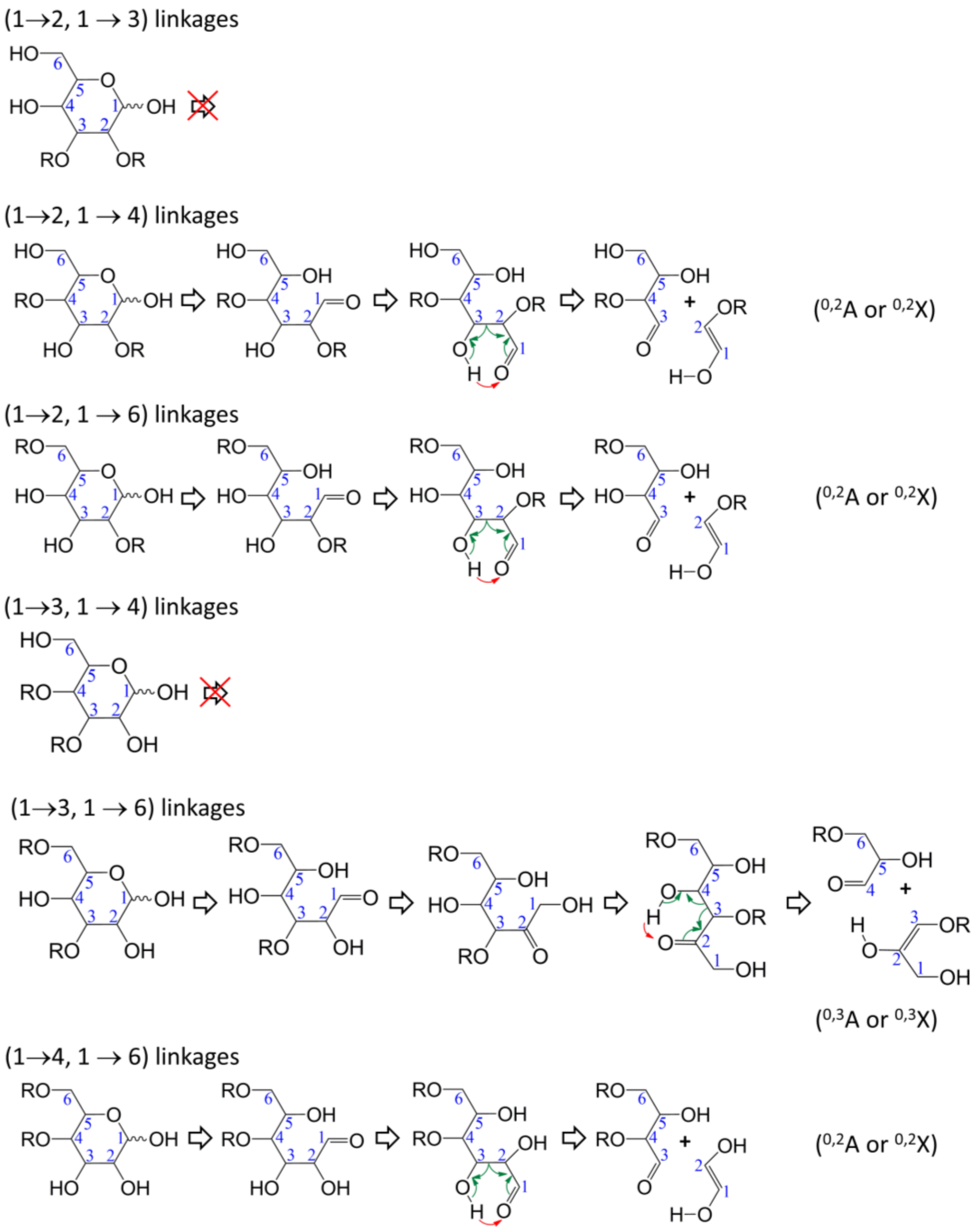

Fig. S2.2 Retro-aldol reaction of the hexose at the reducing end and connects to the other sugars through two glycosidic bonds.

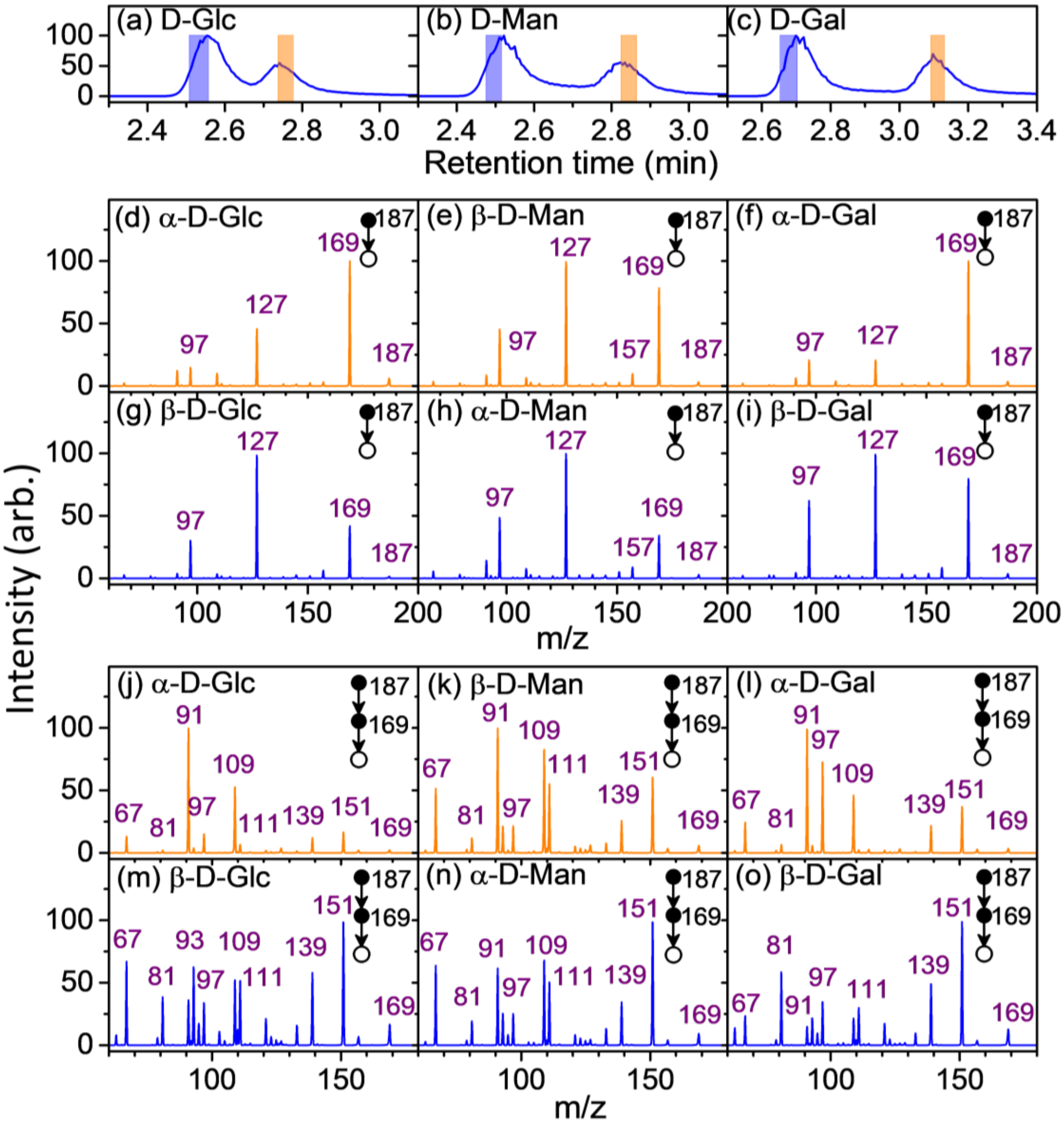

Fig. S2.3. CID spectra of monosaccharide database for the Hex not at the reducing end.

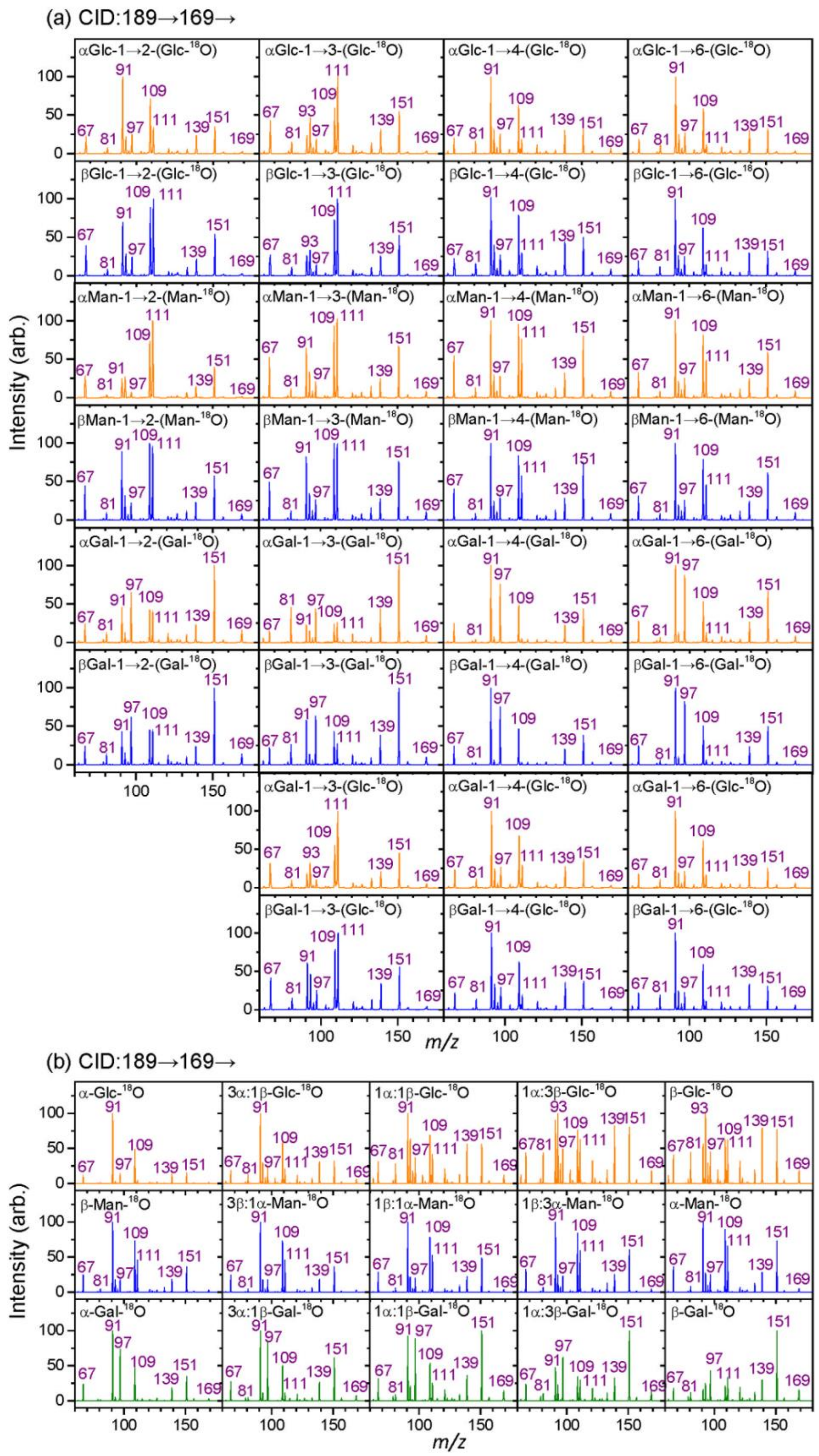

Fig. S2.4. Monosaccharide database for the Hex at the reducing end of oligosaccharide. ^{18}O was labeled at O1 atom of monosaccharides or the sugar at the reducing end of hexose disaccharides. (a) CID spectra of lithium adducts: 351→189→169→fragments. In (b), the CID spectra of lithium adducts:

189→169→fragments of α-Glc-^{18}O, β-Man-^{18}O, α-Gal-^{18}O, β-Glc-^{18}O, α-Man-^{18}O and β-Gal-^{18}O were measured immediately after the separation of two anomers of ^{18}O labeled monosaccharides by HPLC. The other CID spectra were the sum of CID spectra of separated anomers with different ratios (3:1, 1:1, or 1:3).

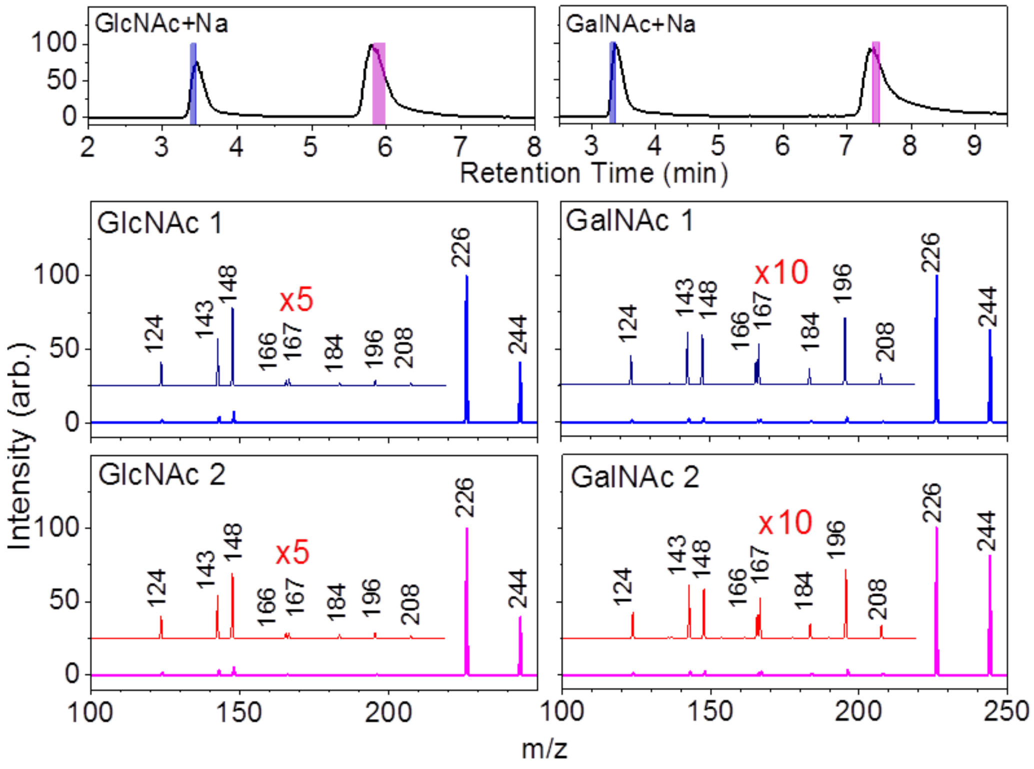

Fig. S2.5. Monosaccharide database for the HexNAc. HPLC chromatograms and CID spectra of HexNAc sodium adducts and the CID spectrum of each anomer measured immediately after the separation of two anomers through HPLC. The blue and pink bars surrounding each peak in the chromatograms represent the period in which the CID spectra were measured. The corresponding spectra are displayed in blue and pink, respectively. These CID spectra were collected to serve as the monosaccharide database.

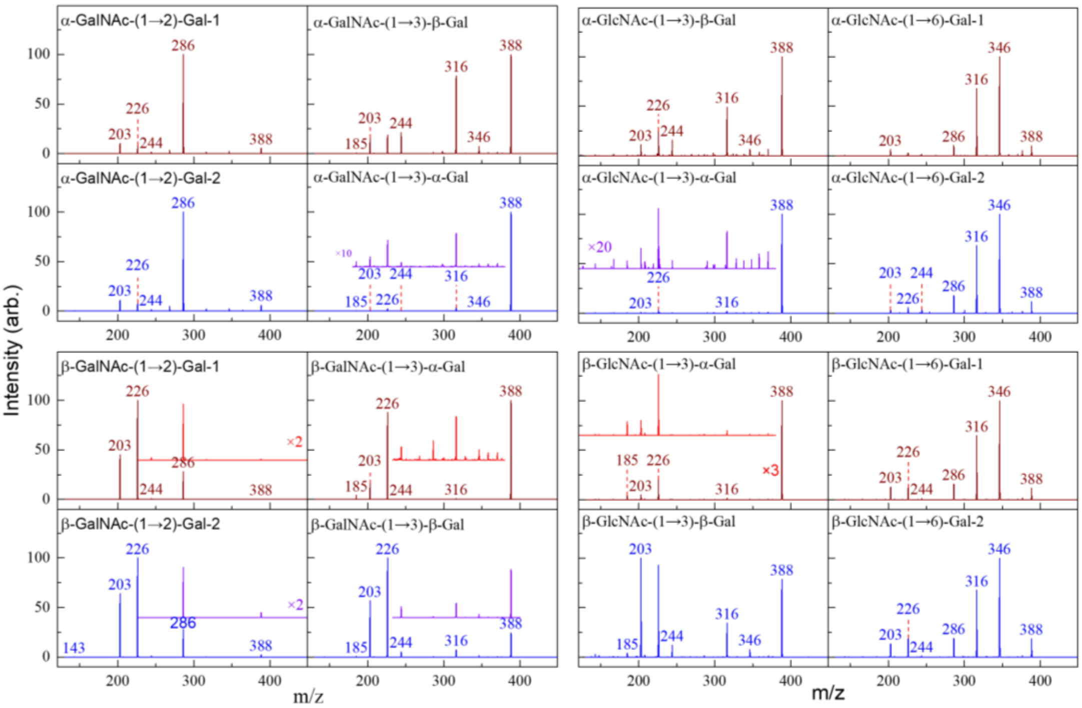

Fig. S2.6 Disaccharide database

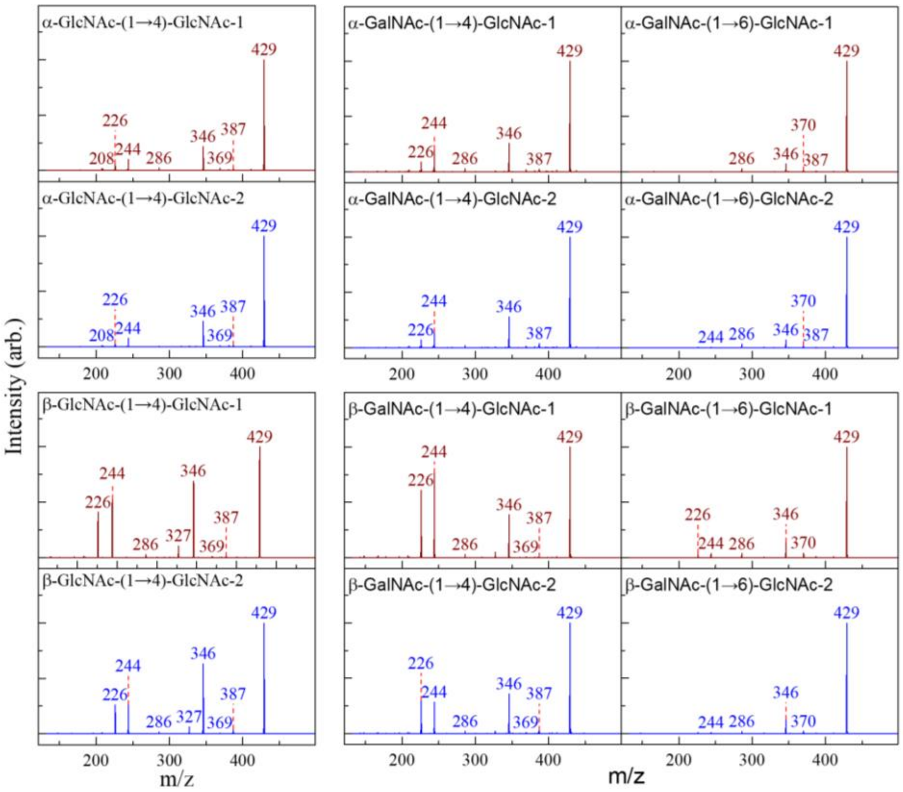

Fig. S2.7 Disaccharide database

3. CID MS^2 of synthesized standards

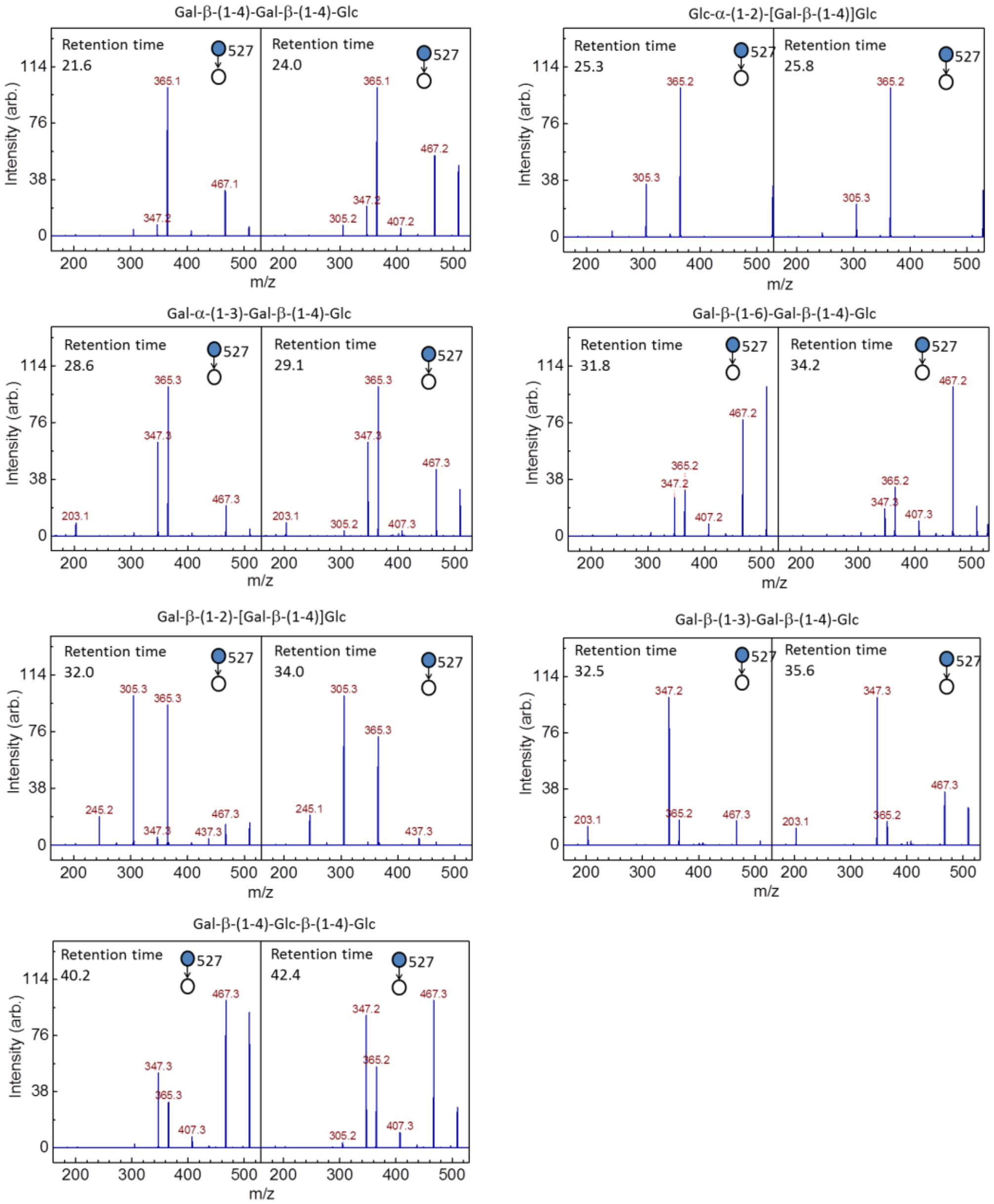

Fig. S3.1. CID MS^2 of synthesized $(Hex)_3$ standards.

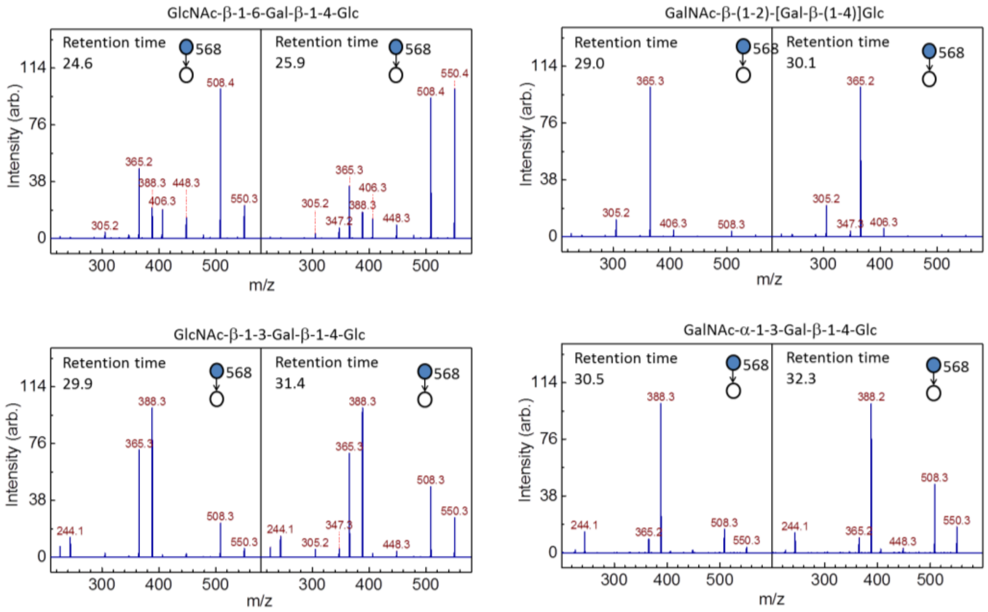

Fig. S3.2. CID MS^2 of synthesized $(Hex)_2$HexNAc standards.

4. Structural analysis using LODES/MS^n

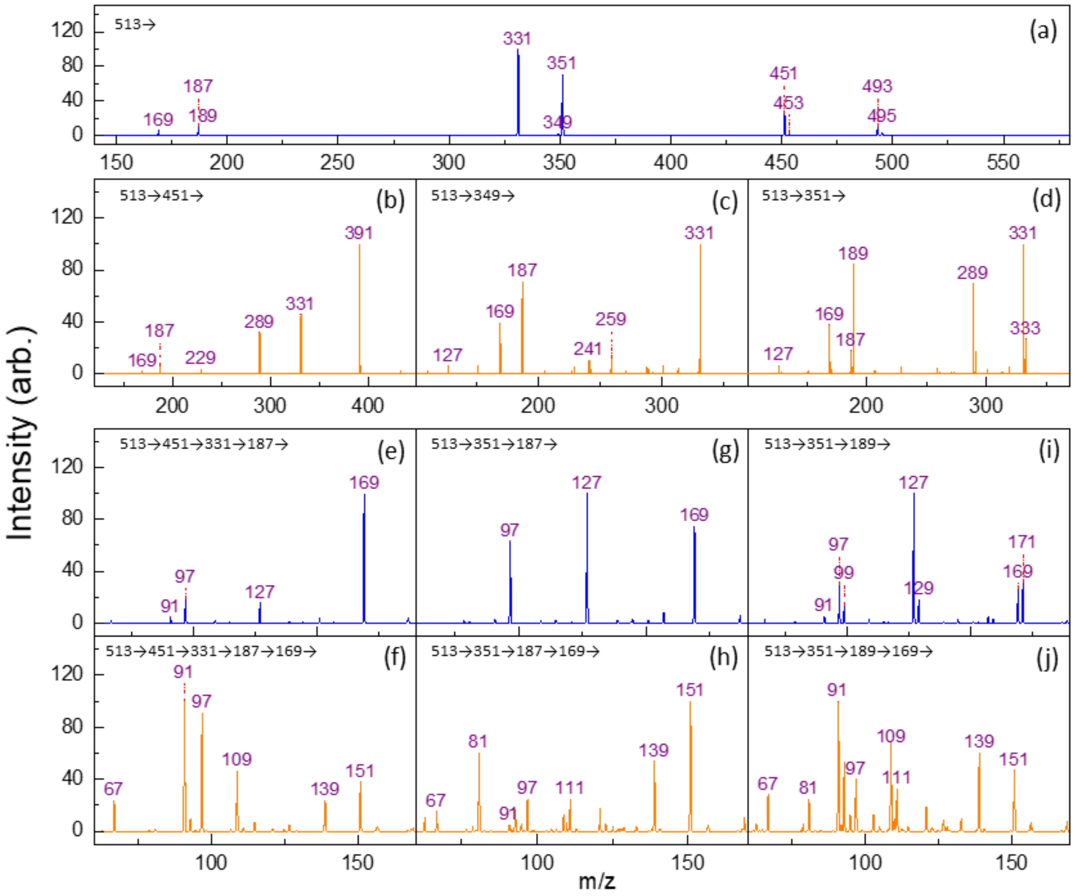

Figure S4.1. CID spectra of ^{18}O labelled (at O1 of the sugar at reducing end) trisaccharide $(Hex)_3$ lithium adduct at retention time 28.9 min in Figure 1(b). Fragment ion *m/z* 451 in (a) indicates the trisaccharide is linear with a linkage of 1→4 at the reducing end or branched with 1→6 and 1→4 linkages at the reducing end. Ion *m/z* 331 in (b) indicates the trisaccharide is linear. Fragment ions *m/z* 259 and *m/z* 289 in (c) and (d), respectively, suggest the linkages at nonreducing end and reducing end are 1→3 and 1→4, respectively. Comparing the CID spectra of (e) and (f), (g) and (h), and (i) and (j) to the CID spectra in monosaccharide database (Figure S2) suggests that the monosaccharides at nonreducing end, center, and reducing end are α-Gal (similarity scores are α-Glc: 74, α-Gal: 94, β-Man: 53), β-Gal (similarity scores are β-Glc: 64, β-Gal: 94, α-Man: 37), and Glc (similarity scores are Glc: 94, Gal: 71, Man: 81), respectively. Calculations of similarity scores are described in our previous report[37]. Consequently, the trisaccharide is determined to be α-Gal-(1→3)-β-Gal-(1→4)-Glc.

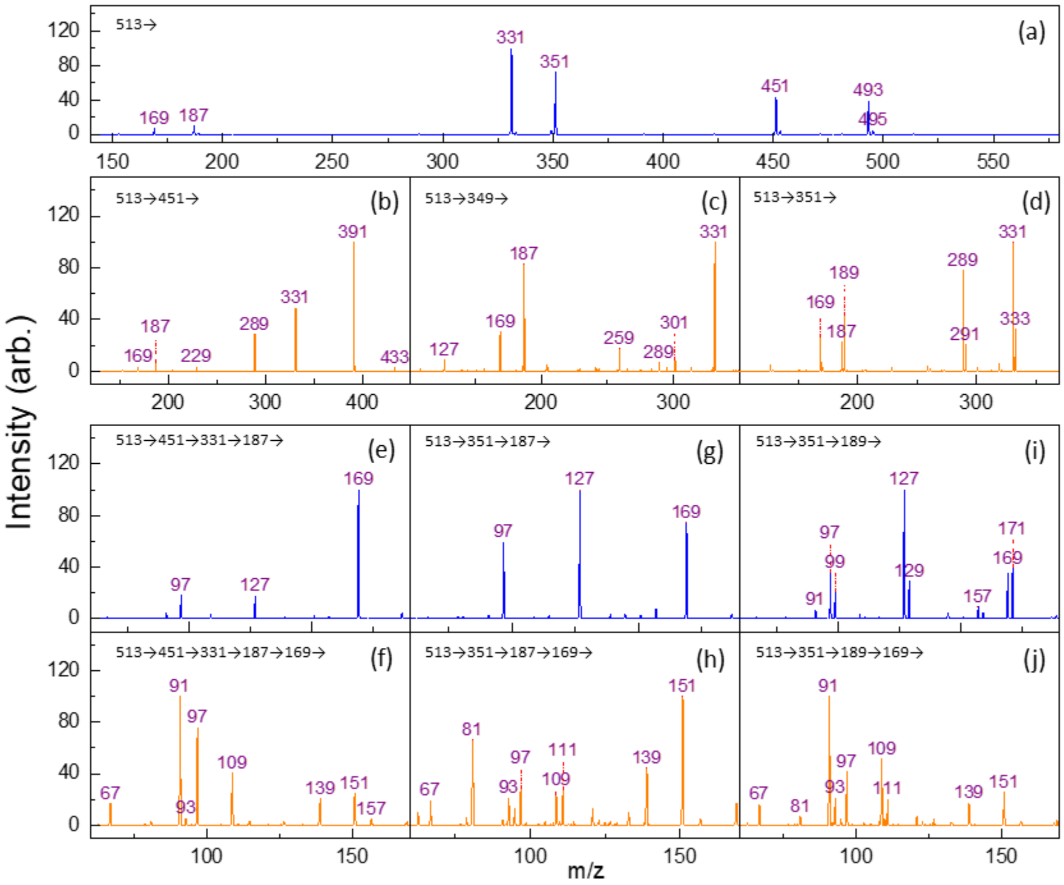

Figure S4.2. CID spectra of ^{18}O labelled (at O1 of the sugar at reducing end) trisaccharide $(Hex)_3$ lithium adduct at retention time 29.3 min in Figure 1(b). Fragment ion *m/z* 451 in (a) indicates the trisaccharide is linear with a linkage of 1→4 at the reducing end or branched with 1→6 and 1→4 linkages at the reducing end. Ion *m/z* 331 in (b) indicates the trisaccharide is linear. Fragment ions *m/z* 259 and *m/z* 289 in (c) and (d), respectively, suggest the linkages at nonreducing end and reducing end are 1→3 and 1→4, respectively. Comparing the CID spectra of (e) and (f), (g) and (h), and (i) and (j) to the CID spectra in monosaccharide database (Figure S2) suggests that the monosaccharides at nonreducing end, center, and reducing end are α-Gal (similarity scores are α-Glc: 79, α-Gal: 97, β-Man: 56), β-Gal (similarity scores are β-Glc: 64, β-Gal: 94, α-Man: 46), and Glc (similarity scores are Glc: 94, Gal: 85, Man: 87), respectively. Calculations of similarity scores are described in our previous report[37]. Consequently, the trisaccharide is determined to be α-Gal-(1→3)-β-Gal-(1→4)-Glc.

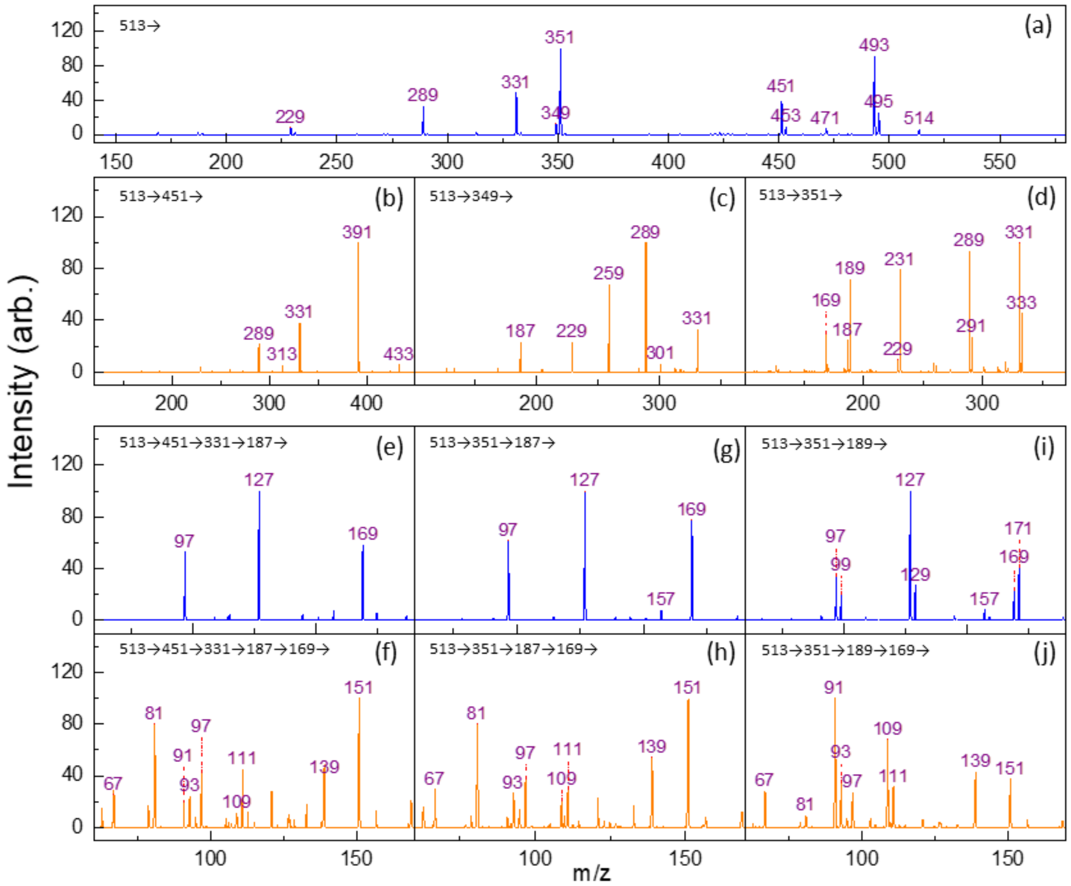

Figure S4.3. CID spectra of ^{18}O labelled (at O1 of the sugar at reducing end) trisaccharide $(Hex)_3$ lithium adduct at retention time 31.8 min in Figure 1(b). Fragment ion *m/z* 451 in (a) indicates the trisaccharide is linear with a linkage of 1→4 at the reducing end or branched with 1→6 and 1→4 linkages at the reducing end. Ion *m/z* 331 in (b) indicates the trisaccharide is linear. Fragment ions *m/z* 229, 259, 289 in (c) and *m/z* 289 in (c) suggest the linkages at nonreducing end and reducing end are 1→6 and 1→4, respectively. Comparing the CID spectra of (e) and (f), (g) and (h), and (i) and (j) to the CID spectra in monosaccharide database (Figure S2) suggests that the monosaccharides at nonreducing end, center, and reducing end are β-Gal (similarity scores are β-Glc: 54, β-Gal: 84, α-Man: 44), β-Gal (similarity scores are β-Glc: 62, β-Gal: 93, α-Man: 43), and Glc (similarity scores are Glc: 95, Gal: 88, Man: 72), respectively. Calculations of similarity scores are described in our previous report[37]. Consequently, the trisaccharide is determined to be β-Gal-(1→6)-β-Gal-(1→4)-Glc. . The CID spectra were taken using the fraction different from that used in Figure 1(b) such that two peaks at 31.8 min were separated.

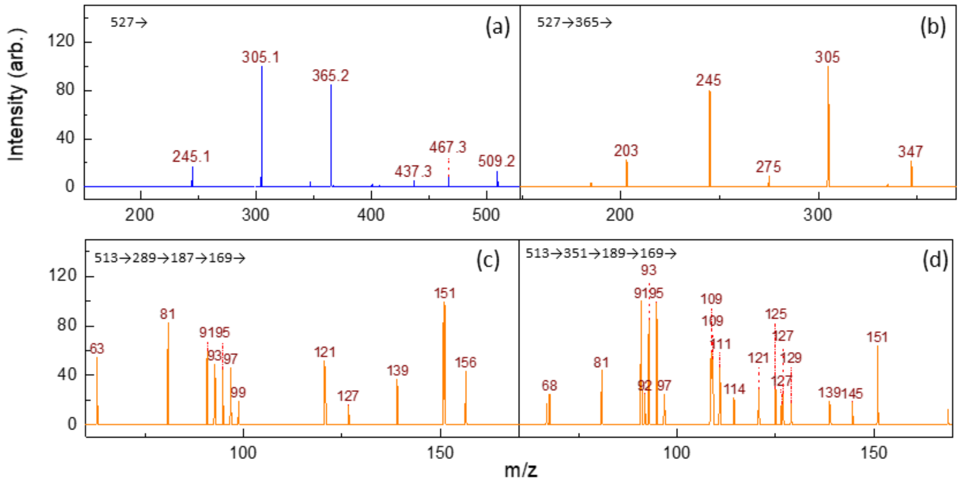

Figure S4.4. CID spectra of $(Hex)_3$ sodium adduct [(a)and (b)] and ^{18}O labelled (at O1 of the sugar at reducing end) $(Hex)_3$ lithium adduct [(c) and (d)] at retention time 31.8 min in Figure 1(b). The high intensity of ion *m/z* 305 in (a) suggest it is a branched trisaccharides with (1→2, 1→4) or (1→2, 1→6) linkages. Ions *m/z* 245 and 305 in (b) suggests the trisaccharide has (1→2, 1→4) linkage. Comparing the spectra in (c) and (d) to the monosaccharide database in Figure S2 suggests the hexose at the nonreducing end which connecting to 1→4 linkage is β-Gal (similarity scores are β-Glc: 70, β-Gal: 86, α-Man: 75), but the hexose at the reducing end cannot be determined due to similar similarity scores (similarity scores are Glc: 83, Gal: 62, Man: 84). The hexose at the nonreducing end connecting to 1→2 linkage cannot be determined because the signal is small due to low abundance. The rest of structural determination is made by comparison to the chromatogram retention time of chemically synthesized β-Gal-(1→4)-[β-Gal-(1→2)]-Glc, as illustrated in Figure S3.1. The CID spectra were taken using the fraction different from that used in Figure 1(b) such that two peaks at 31.8 min were separated.

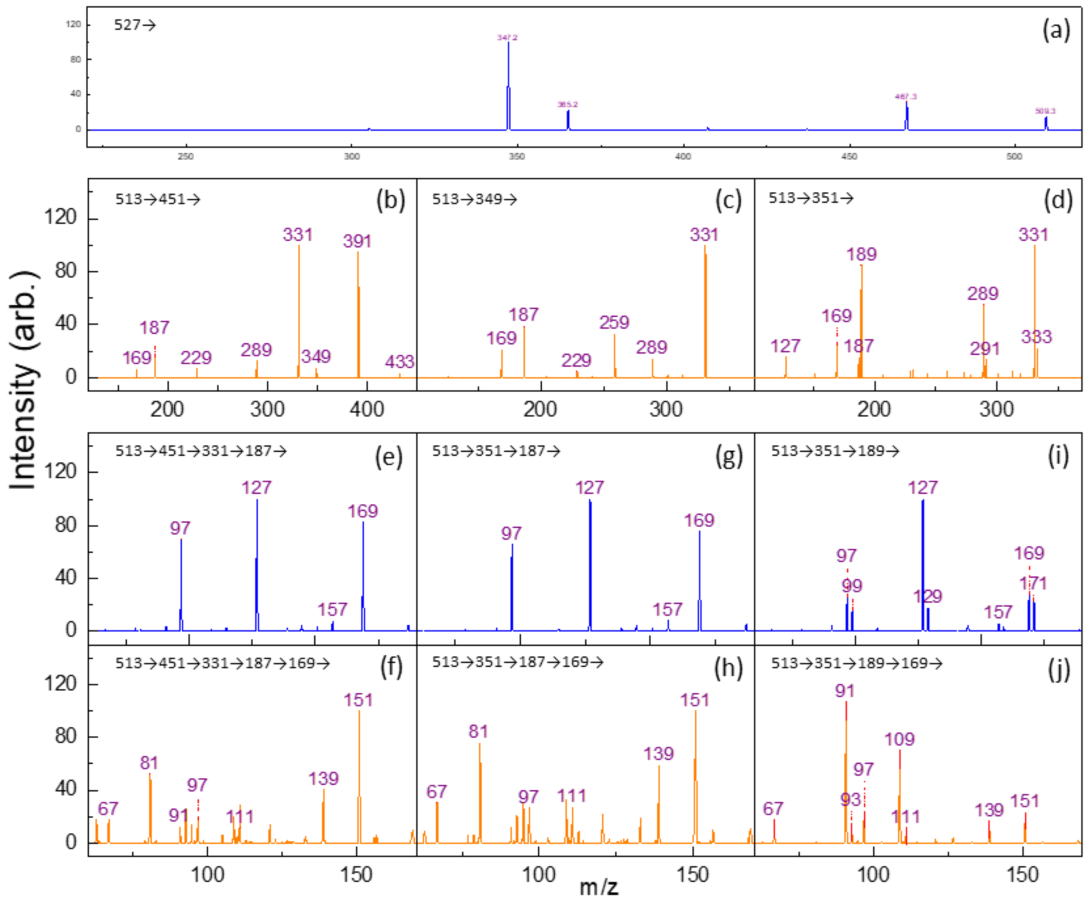

Figure S4.5. CID spectra of trisaccharide (Hex)$_3$ sodium adduct (a) and ^{18}O labelled (at O1 of the sugar at reducing end) lithium adduct (b)-(j) at retention time 32.6 min in Figure 1(b). Fragment ion *m/z* 467 in (a) indicates the trisaccharide is linear with a linkage of 1→4 at the reducing end or branched with 1→6 and 1→4 linkages at the reducing end. Ion *m/z* 331 in (b) indicates the trisaccharide is linear. Fragment ions *m/z* 259 and *m/z* 289 in (c) and (d), respectively, suggest the linkages at nonreducing end and reducing end are 1→3 and 1→4, respectively. Comparing the CID spectra of (e) and (f), (g) and (h), and (i) and (j) to the CID spectra in monosaccharide database (Figure S2) suggests that the monosaccharides at nonreducing end, center, and reducing end are β-Gal (similarity scores are β-Glc: 70, β-Gal: 95, α-Man: 39), β-Gal (similarity scores are β-Glc: 62, β-Gal: 93, α-Man: 51), and Glc (similarity scores are Glc: 96, Gal: 81, Man: 87), respectively. Calculations of similarity scores are described in our previous report[37]. Consequently, the trisaccharide is determined to be β-Gal-(1→3)-β-Gal- (1→4)-Glc.

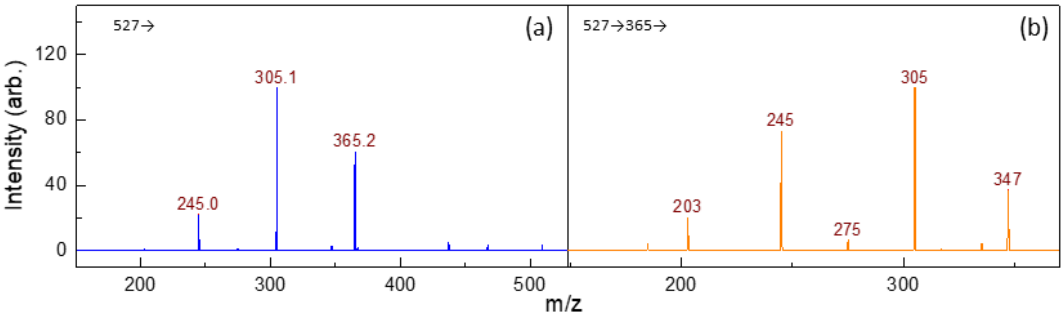

Figure S4.6. CID spectra of trisaccharide $(Hex)_3$ sodium adduct [(a) and (b)] at retention time 34.1 min in Figure 1(b). Fragment ions *m/z* 305 and 245 in (a) indicate the trisaccharide is branched with (1→6, 1→2) or (1→4, 1→2) linkages. Fragments ion *m/z* 305 and 245 in (b) indicate the linkages are (1→4, 1→2). The hexose cannot be determined because the small signal. The rest of structural determination is made by the CID of the other anomer (at 31.8 min) and the comparison to the chromatogram retention time of chemically synthesized β-Gal-(1→4)-[β-Gal-(1→2)]-Glc in Figure S3.1.

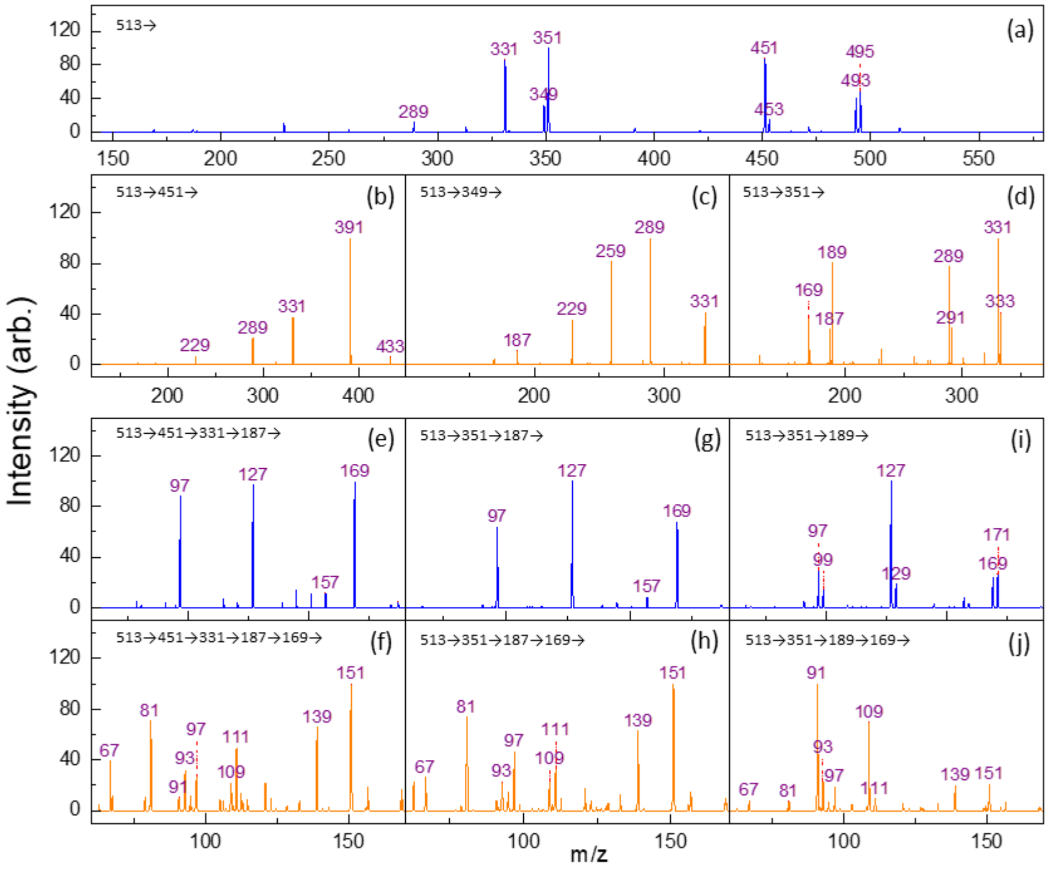

Figure S4.7. CID spectra of ^{18}O labelled (at O1 of the sugar at reducing end) trisaccharide $(Hex)_3$ lithium adduct at retention time 34.5 min in Figure 1(b). Fragment ion *m/z* 451 in (a) indicates the trisaccharide is linear with a linkage of 1→4 at the reducing end or branched with 1→6 and 1→4 linkages at the reducing end. Ion *m/z* 331 in (b) indicates the trisaccharide is linear. Fragment ions *m/z* 289, 259, and 229 in (c) and ion *m/z* 289 in (d), respectively, suggest the linkages at nonreducing end and reducing end are 1→6 and 1→4, respectively. Comparing the CID spectra of (e) and (f), (g) and (h), and (i) and (j) to the CID spectra in monosaccharide database (Figure S2) suggests that they are β-Gal (similarity scores are β-Glc: 63, β-Gal: 93, α-Man: 45), β-Gal (similarity scores are β-Glc: 56, β-Gal: 87, α-Man: 50), and Glc (similarity scores are Glc: 95, Gal: 81, Man: 90), respectively. Consequently, the trisaccharide is determined to be β-Gal-(1→6)-β-Gal-(1→4)-Glc.

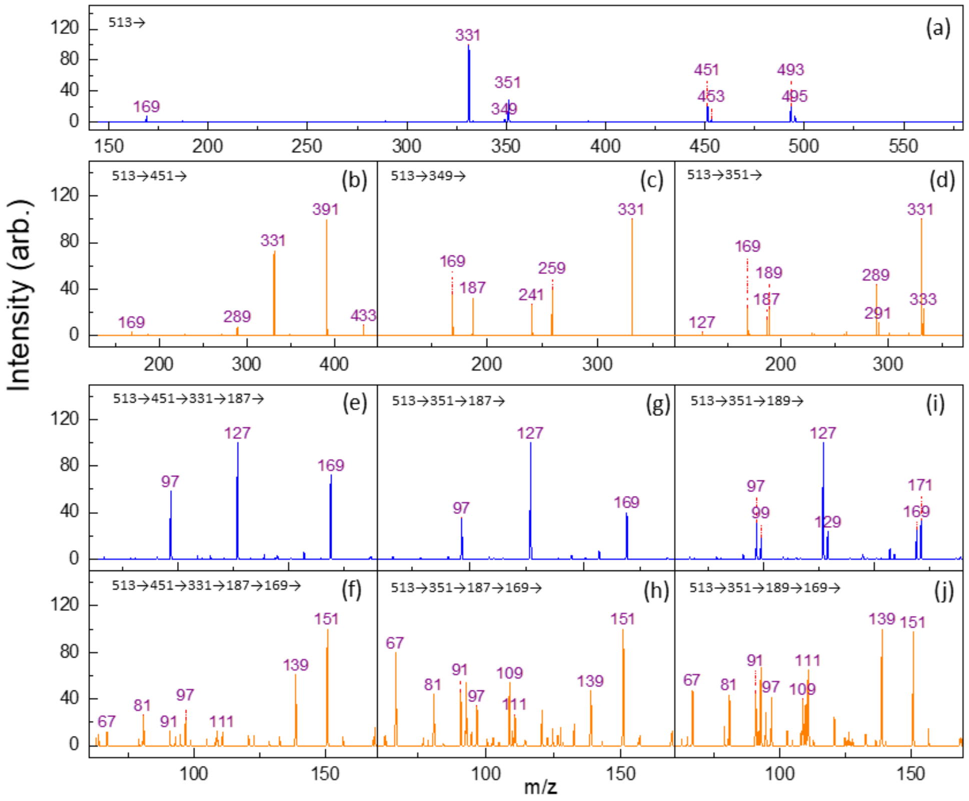

Figure S4.8. CID spectra of ^{18}O labelled (at O1 of the sugar at reducing end) trisaccharide $(Hex)_3$ lithium adduct at retention time 35.7 min in Figure 1(b). Fragment ion *m/z* 451 in (a) indicates the trisaccharide is linear with a linkage of 1→4 at the reducing end or branched with 1→6 and 1→4 linkages at the reducing end. Ion *m/z* 331 in (b) indicates the trisaccharide is linear. Fragment ions *m/z* 259 and *m/z* 289 in (c) and (d), respectively, suggest the linkages at nonreducing end and reducing end are 1→3 and 1→4, respectively. Comparing the CID spectra of (e) and (f), (g) and (h), and (i) and (j) to the CID spectra in monosaccharide database (Figure S2) suggests that they are β-Gal (similarity scores are β-Glc: 60, β-Gal: 90, α-Man: 44), β-Gal (similarity scores are β-Glc: 67, β-Gal: 94, α-Man: 37), and Glc (similarity scores are Glc: 92, Gal: 80, Man: 91), respectively. Consequently, the trisaccharide is determined to be β-Gal-(1→3)-β-Gal-(1→4)-Glc. The hexose at the reducing end has close similarity scores of Glc and Man. The stereoisomer of the hexose at the reducing end is crossed checked using another anomer of this isomer at retention time 32.6 min.

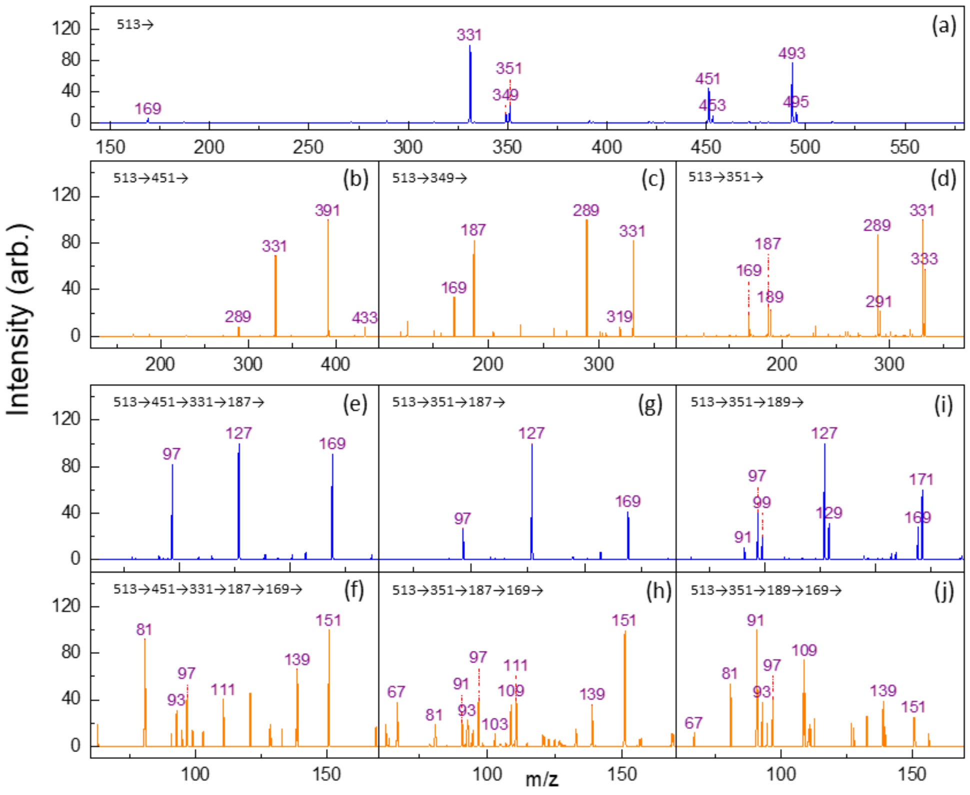

Figure S4.9. CID spectra of ^{18}O labelled (at O1 of the sugar at reducing end) trisaccharide $(Hex)_3$ lithium adduct at retention time 40.1 min in Figure 1(b). Fragment ion *m/z* 451 in (a) indicates the trisaccharide is linear with a linkage of 1→4 at the reducing end or branched with 1→6 and 1→4 linkages at the reducing end. Ion *m/z* 331 in (b) indicates the trisaccharide is linear. Fragment ion *m/z* 289 in (c) and (d) suggests the linkages at nonreducing end and reducing end are both 1→4. Comparing the CID spectra of (e) and (f), (g) and (h), and (i) and (j) to the CID spectra in monosaccharide database (Figure S2) suggests that they are β-Gal (similarity scores are β-Glc: 88, β-Gal: 96, α-Man: 61), β-Glc (similarity scores are β-Glc: 93, β-Gal: 66, α-Man: 79), and Glc (similarity scores are Glc: 94, Gal: 79, Man: 88), respectively. Consequently, the trisaccharide is determined to be β-Gal-(1→4)-β-Glc-(1→4)-Glc.

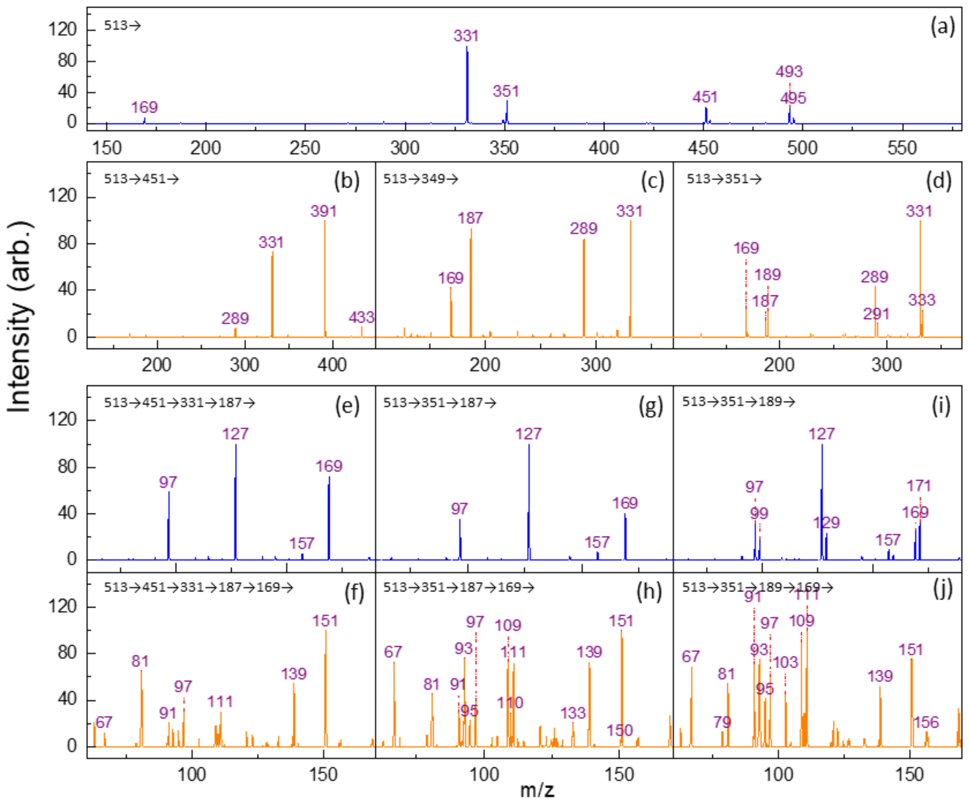

Figure S4.10. CID spectra of [18]O labelled (at O1 of the sugar at reducing end) trisaccharide $(Hex)_3$ lithium adduct at retention time 42.2 min in Figure 1(b). Fragment ion *m/z* 451 in (a) indicates the trisaccharide is linear with a linkage of 1→4 at the reducing end or branched with 1→6 and 1→4 linkages at the reducing end. Ion *m/z* 331 in (b) indicates the trisaccharide is linear. Fragment ion *m/z* 289 in (c) and (d) suggests the linkages at nonreducing end and reducing end are both 1→4. Comparing the CID spectra of (e) and (f), (g) and (h), and (i) and (j) to the CID spectra in monosaccharide database (Figure S2) suggests that they are β-Gal (similarity scores are β-Glc: 60, β-Gal: 91, α-Man: 47), β-Glc (similarity scores are β-Glc: 97, β-Gal: 70, α-Man: 69), and Glc (similarity scores are Glc: 83, Gal: 57, Man: 69), respectively. Consequently, the trisaccharide is determined to be β-Gal-(1→4)-β-Glc-(1→4)-Glc.

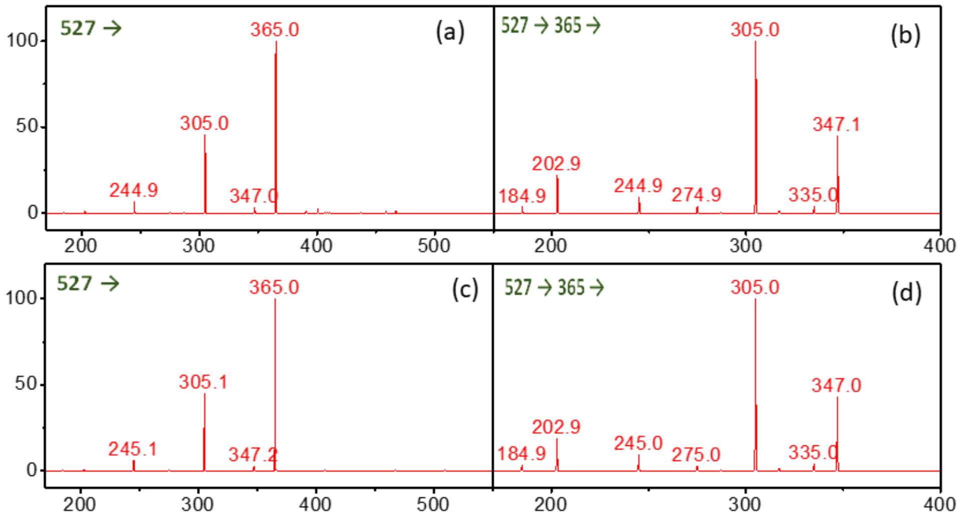

Figure S4.11. CID spectra of trisaccharide $(Hex)_3$ sodium adduct (a) and (b) at retention time 25.3 min in Figure 1(c). No fragments of *m/z* 467, 437, 407 and fragment ion *m/z* 305 and 245 in (a) indicates the trisaccharide is branched with (1→2, 1→4) or (1→2, 1→6) linkages. Ions *m/z* 305 and 245 in (b) indicates the trisaccharide is branched with (1→2, 1→4) linkage. Due to the low abundance of this trisaccharide, we did not identify the anomericity and stereoisomer. We compared to the CID spectra of the chemically synthesized trisaccharide α-Glc-(1→2)-[β-Gal-(1→4)]-Glc, (c) and (d), in the retention time 25.8 min in Figure S3.1. The same retention time and similar CID spectra indicate the trisaccharide is α-Glc-(1→2)-[β-Gal-(1→4)]-Glc.

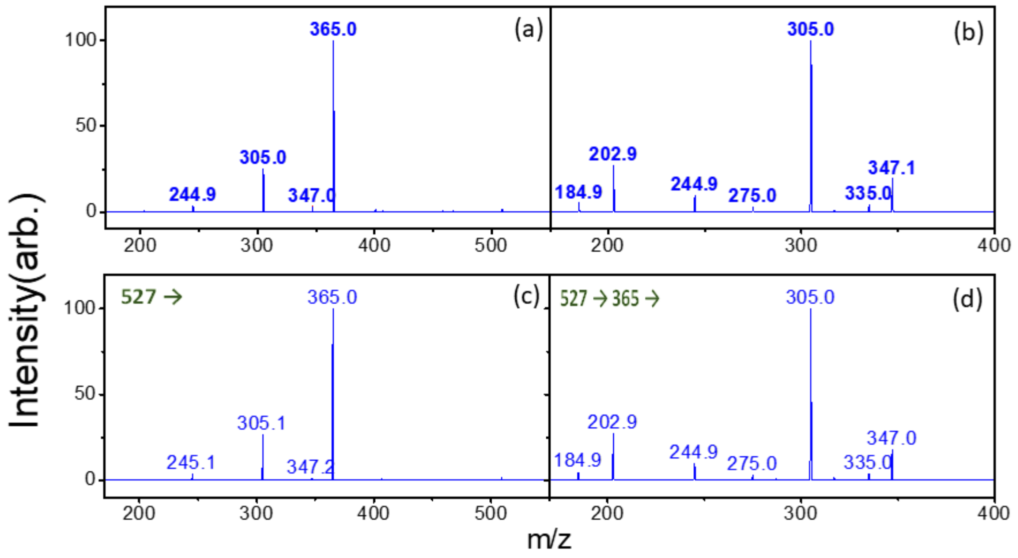

Figure S4.12. CID spectra of trisaccharide $(Hex)_3$ sodium adduct (a) and (b) at retention time 25.8 min in Figure 1(c). No fragments of *m/z* 467, 437, 407 and fragment ion *m/z* 305 and 245 in (a) indicates the trisaccharide is branched with (1→2, 1→4) or (1→2, 1→6) linkages. Ions *m/z* 305 and 245 in (b) indicates the trisaccharide is branched with (1→2, 1→4) linkage. Due to the low abundance of this trisaccharide, we did not identify the anomericity and stereoisomer. We compared to the CID spectra of the chemically synthesized trisaccharide α-Glc-(1→2)-[β-Gal-(1→4)]-Glc, (c) and (d), in the retention time 25.8 min in Figure S3.1. The same retention time and similar CID spectra indicate the trisaccharide is α-Glc-(1→2)-[β-Gal-(1→4)]-Glc.

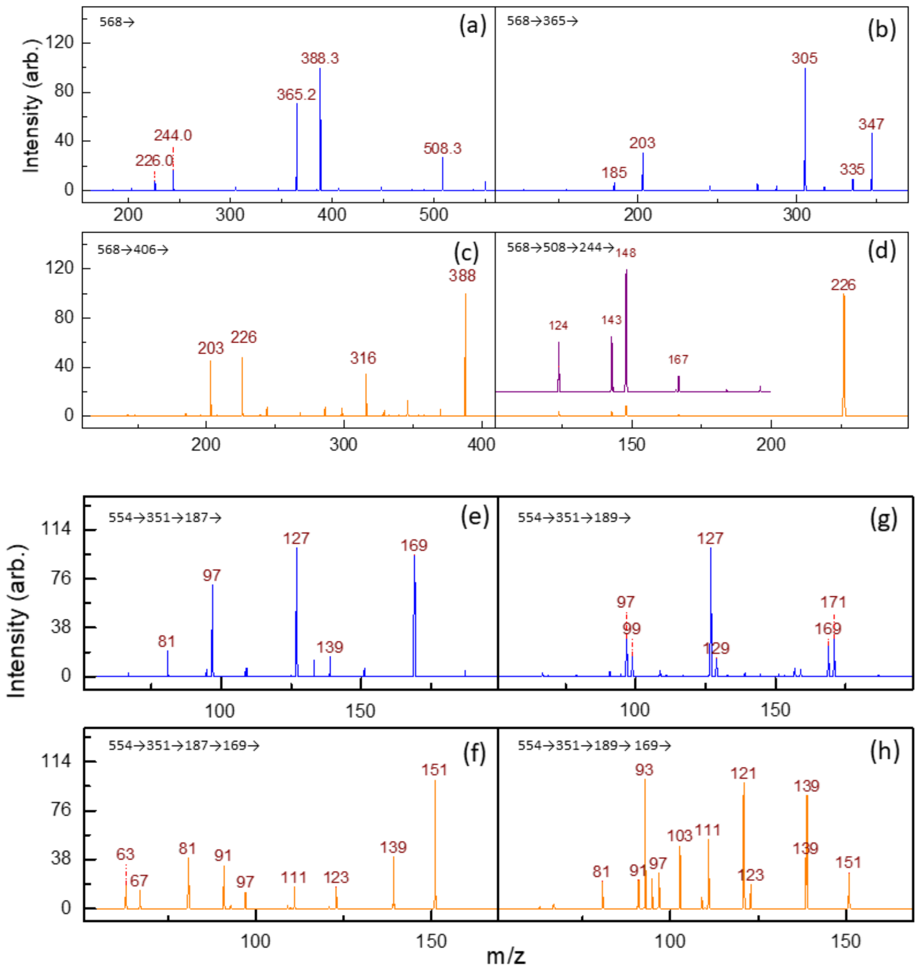

Figure S4.13. CID spectra of trisaccharide $(Hex)_2HexNAc$ sodium adduct (a)-(e) and ^{18}O labelled (at O1 of the sugar at reducing end) lithium adduct (f)-(i) at retention time 29.9 min in Figure 1(d). Fragment ion *m/z* 508 in (a) indicates the trisaccharide is linear with a linkage of 1→4 at the reducing end or branched with 1→6 and 1→4 linkages at the reducing end. Ion *m/z* 305 in (b) indicates the linkage between HexNAc-Hex is 1→3, and ion *m/z* 316 in (c) indicates the linkage between Hex-Hex is 1→4. Comparing the CID spectra of (d), (e) and (f), and (g) and (h) to the CID spectra in monosaccharide database (Figure S2) suggests HexNAc, hexose at center, and hexose at reducing end are GlcNAc, β-Gal (similarity scores are β-Glc: 49, β-Gal: 79, α-Man: 42), and Glc (similarity scores are Glc: 63, Gal: 61, Man: 54), respectively. Comparing (d) to disaccharide database GlcNAc-(1→3)-Gal in Figure S2.6 suggests the linkage is β-1→3. Consequently, the trisaccharide is determined to be β-GlcNAc-(1→3)-β-Gal-(1→4)-Glc. The close similarity scores of Glc and Gal of the hexose at the reducing end is double checked using the CID spectra at retention time 31.3 min in Figure 1(d).

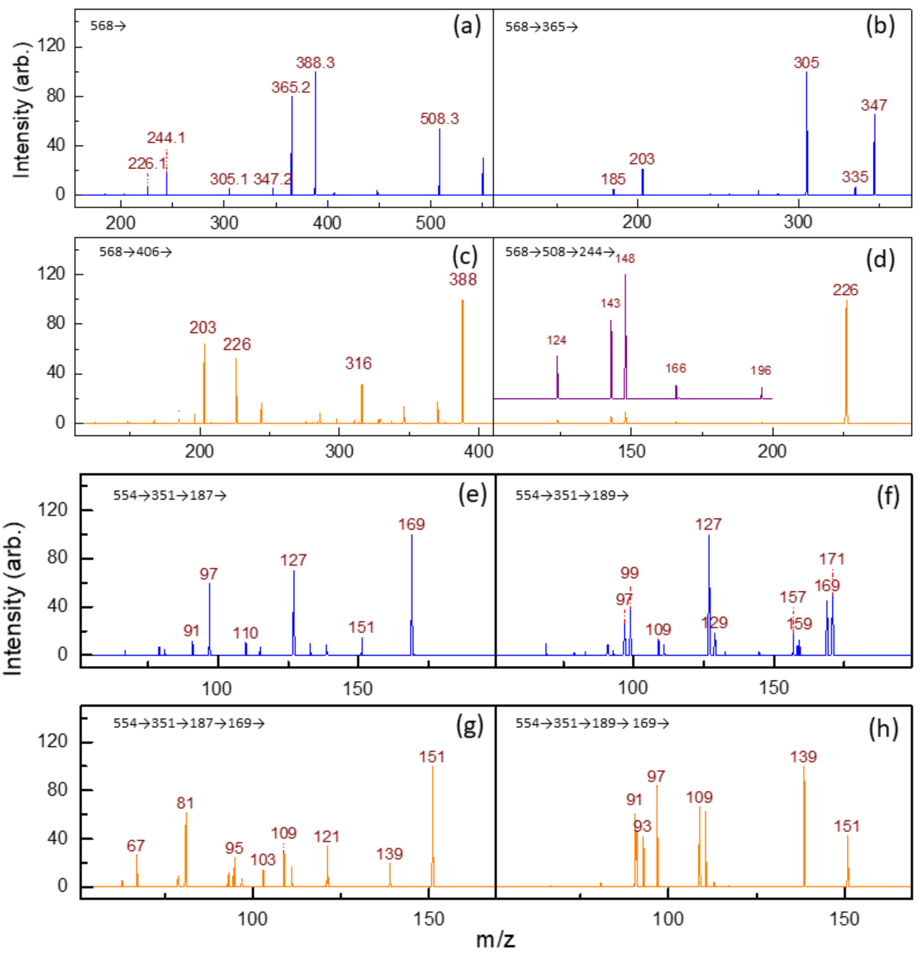

Figure S4.14. CID spectra of trisaccharide $(Hex)_2HexNAc$ sodium adduct (a)-(e) and ^{18}O labelled (at O1 of the sugar at reducing end) lithium adduct (f)-(i) at retention time 31.3 min in Figure 1(d). Fragment ion *m/z* 508 in (a) indicates the trisaccharide is linear with a linkage of 1→4 at the reducing end or branched with 1→6 and 1→4 linkages at the reducing end. Ion *m/z* 305 in (b) indicates the linkage between HexNAc-Hex is 1→3, and ion *m/z* 316 in (c) indicates the linkage between Hex-Hex is 1→4. Comparing the CID spectra of (d), (e) and (f), and (g) and (h) to the CID spectra in monosaccharide database (Figure S2) suggests HexNAc, hexose at center, and hexose at reducing end are GlcNAc, β-Gal (similarity scores are β-Glc: 43, β-Gal: 73, α-Man: 42), and Glc (similarity scores are Glc: 88 Gal: 70, Man: 80), respectively. Comparing (d) to disaccharide database GlcNAc-(1→3)-Gal in Figure S2.6 suggests the linkage is β-1→3. Consequently, the trisaccharide is determined to be β-GlcNAc-(1→3)-β-Gal-(1→4)-Glc.

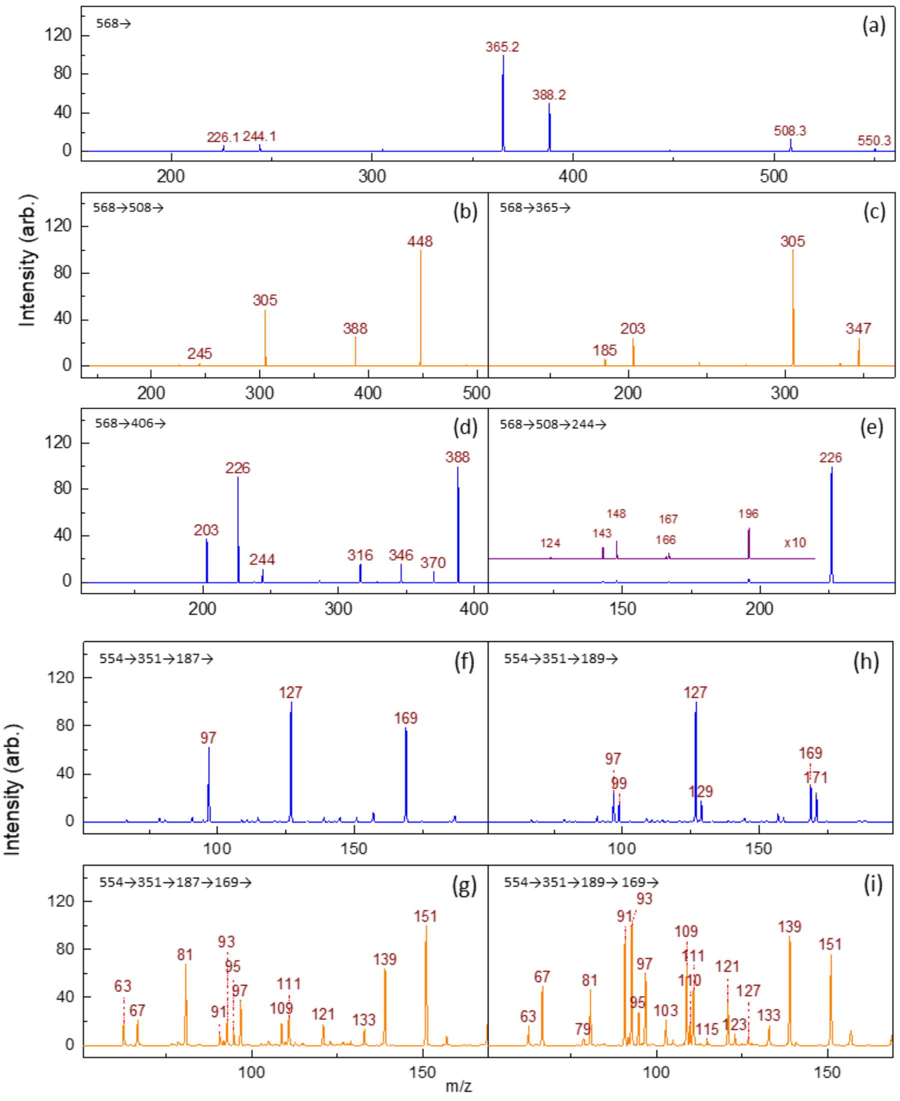

Figure S4.15. CID spectra of trisaccharide $(Hex)_2HexNAc$ sodium adduct (a)-(e) and ^{18}O labelled (at O1 of the sugar at reducing end) lithium adduct (f)-(i) at retention time 27.1 min in Figure 1(e). Fragment ion *m/z* 508 in (a) indicates the trisaccharide is linear with a linkage of 1→4 at the reducing end or branched with 1→6 and 1→4 linkages at the reducing end. Ion *m/z* 388 in (b) indicates the trisaccharide is linear. Comparing the CID spectra of (e), (f) and (g), and (h) and (i) to the CID spectra in monosaccharide database (Figure S2) suggests that HexNAc, hexose at the nonreducing end, and hexose at the reducing end are GalNAc, β-Gal (similarity scores are β-Glc: 68, β-Gal: 93, α-Man: 36), and Glc (similarity scores are Glc: 89, Gal: 61, Man: 62), respectively. Fragment ion *m/z* 305 in (c) and *m/z* 316 in (d) suggests the linkages at reducing end and nonreducing end are 1→4 and 1→3, respectively. Comparing (d) to disaccharide database GalNAc-(1→3)-Gal in Figure S2.6 suggests the linkage in (d) is β-1→3. Consequently, the trisaccharide is determined to be β-GalNAc-(1→3)-β-Gal-(1→4)-Glc.

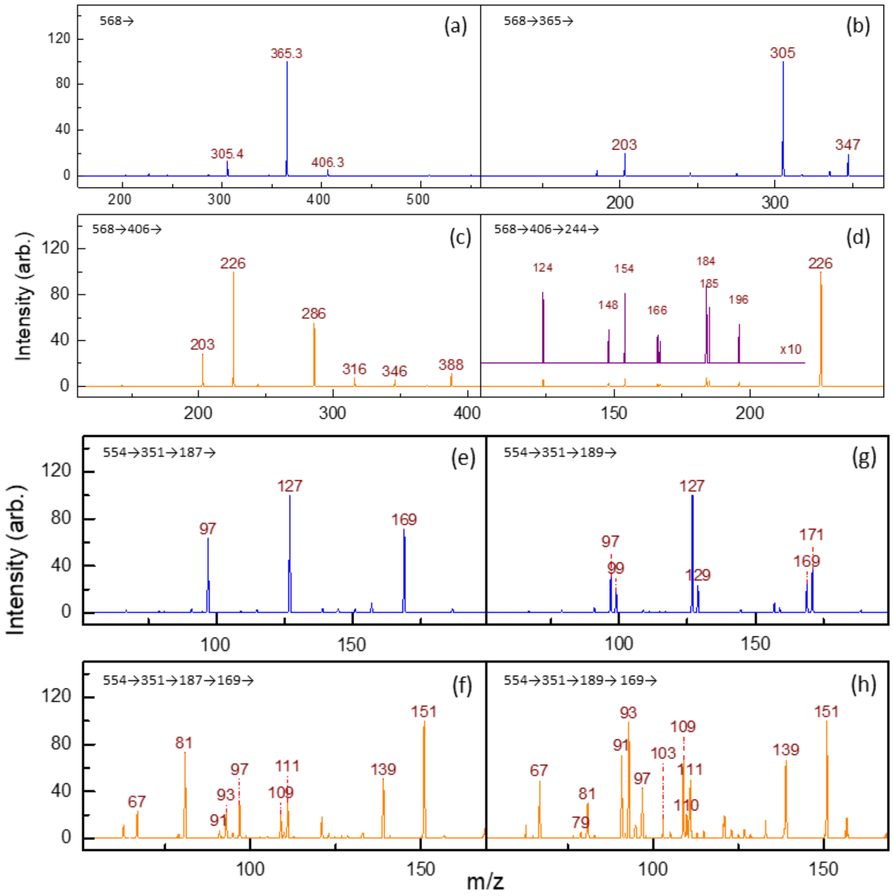

Figure S4.16. CID spectra of trisaccharide $(Hex)_2$HexNAc sodium adduct (a)-(d) and ^{18}O labelled (at O1 of the sugar at reducing end) lithium adduct (e)-(h) at retention time 28.9 min in Figure 1(e). Fragment ion *m/z* 305 in (a) indicates the trisaccharide is branched with (1→6, 1→2) or (1→4, 1→2) linkages. Ion *m/z* 305 in (b) and *m/z* 286 in (c) indicates the linkages between Hex-Hex and HexNAc-Hex are 1→4 and 1→2, respectively. Comparing the CID spectra of (d), (e) and (f), and (g) and (h) to the CID spectra in monosaccharide database (Figure S2) suggests HexNAc, hexose at nonreducing end, and hexose at reducing end are GalNAc, β-Gal (similarity scores are β-Glc: 64, β-Gal: 94, α-Man: 44), and Glc (similarity scores are Glc: 96, Gal: 60, Man: 67), respectively. Comparing (c) to disaccharide database GalNAc-(1→2)-Gal in Figure S2.6 suggests the linkage is β-1→2. Consequently, the trisaccharide is determined to be β-Glc-(1→4)-[β-GalNAc-(1→2)]-Glc.

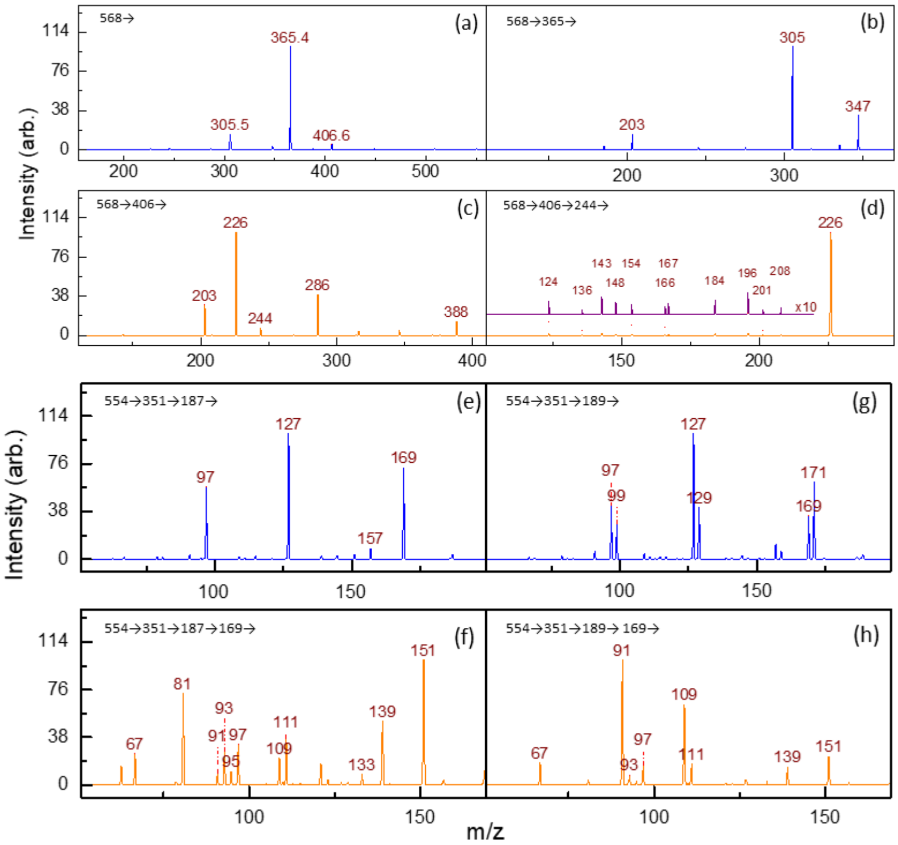

Figure S4.17. CID spectra of trisaccharide (Hex)$_2$HexNAc sodium adduct (a)-(d) and ^{18}O labelled (at O1 of the sugar at reducing end) lithium adduct (e)-(h) at retention time 29.8 min in Figure 1(e). Fragment ion *m/z* 305 in (a) indicates the trisaccharide is branched with (1→6, 1→2) or (1→4, 1→2) linkages. Ion *m/z* 305 in (b) and *m/z* 286 in (c) indicates the linkages between Hex-Hex and HexNAc-Hex are 1→4 and 1→2, respectively. Comparing the CID spectra of (d), (e) and (f), and (g) and (h) to the CID spectra in monosaccharide database (Figure S2) suggests HexNAc, hexose at nonreducing end, and hexose at reducing end are GalNAc, β-Gal (similarity scores are β-Glc: 65, β-Gal: 96, α-Man: 43), and Glc (similarity scores are Glc: 93, Gal: 81, Man: 91), respectively. Comparing (c) to disaccharide database GalNAc-(1→2)-Gal in Figure S2.6 suggests the linkage is β-1→2. Consequently, the trisaccharide is determined to be β-Glc-(1→4)-[β-GalNAc-(1→2)]-Glc.

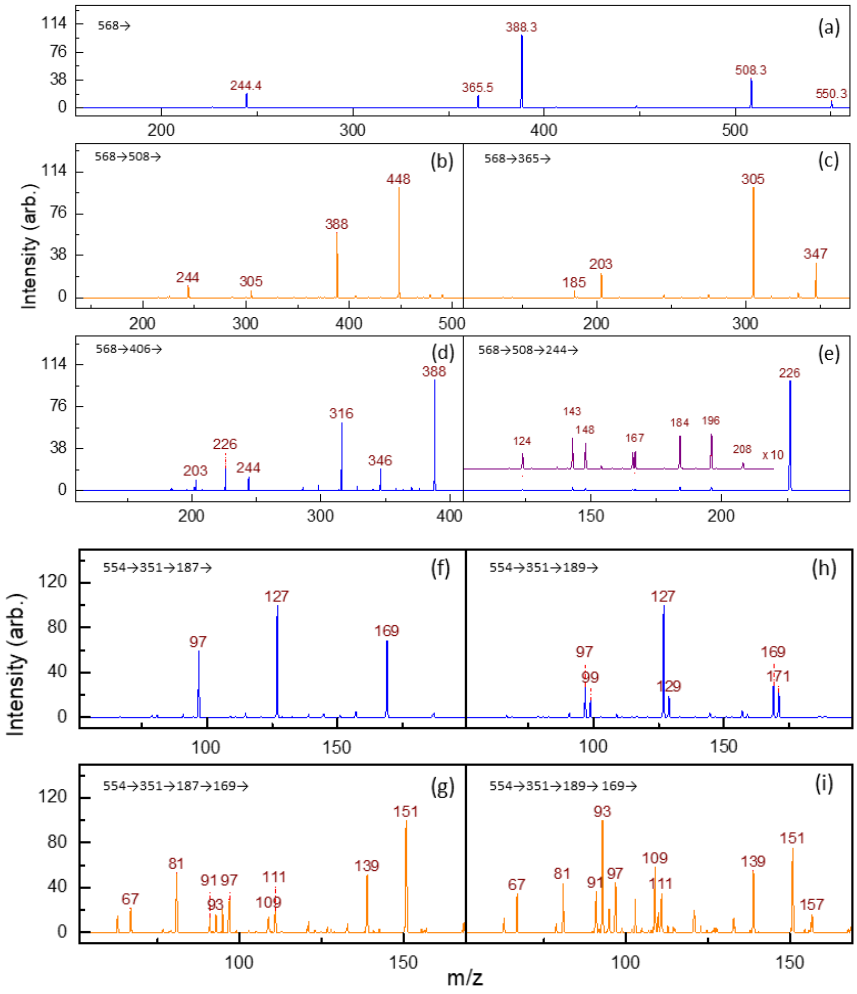

Figure S4.18. CID spectra of trisaccharide $(Hex)_2HexNAc$ sodium adduct (a)-(e) and ^{18}O labelled (at O1 of the sugar at reducing end) lithium adduct (f)-(i) at retention time 30.6 min in Figure 1(e). Fragment ion *m/z* 508 in (a) indicates the trisaccharide is linear with a linkage of 1→4 at the reducing end or branched with 1→6 and 1→4 linkages at the reducing end. Ion *m/z* 388 in (b) indicates the trisaccharide is linear. Ion *m/z* 305 in (c) and ion *m/z* 316 in (d) indicate the linkages of Hex-Hex and HexNAc-Hex are 1→4 and 1→3, respectively. Comparing the CID spectra of (e), (f) and (g), and (h) and (i) to the CID spectra in monosaccharide database (Figure S2) suggests HexNAc, hexose at center, and hexose at reducing end are GalNAc, β-Gal (similarity scores are β-Glc: 68, β-Gal: 94, α-Man: 37), and Glc (similarity scores are Glc: 87, Gal: 70, Man: 62), respectively. Fragment ion *m/z* 305 in (c) and *m/z* 316 in (d) suggests the linkages at reducing end and nonreducing end are 1→4 and 1→3, respectively. Comparing (d) to disaccharide database GalNAc-(1→3)-Gal in Figure S2.6 suggests the linkage is α-1→3. Consequently, the trisaccharide is determined to be α-GalNAc-(1→3)-β-Gal-(1→4)-Glc.

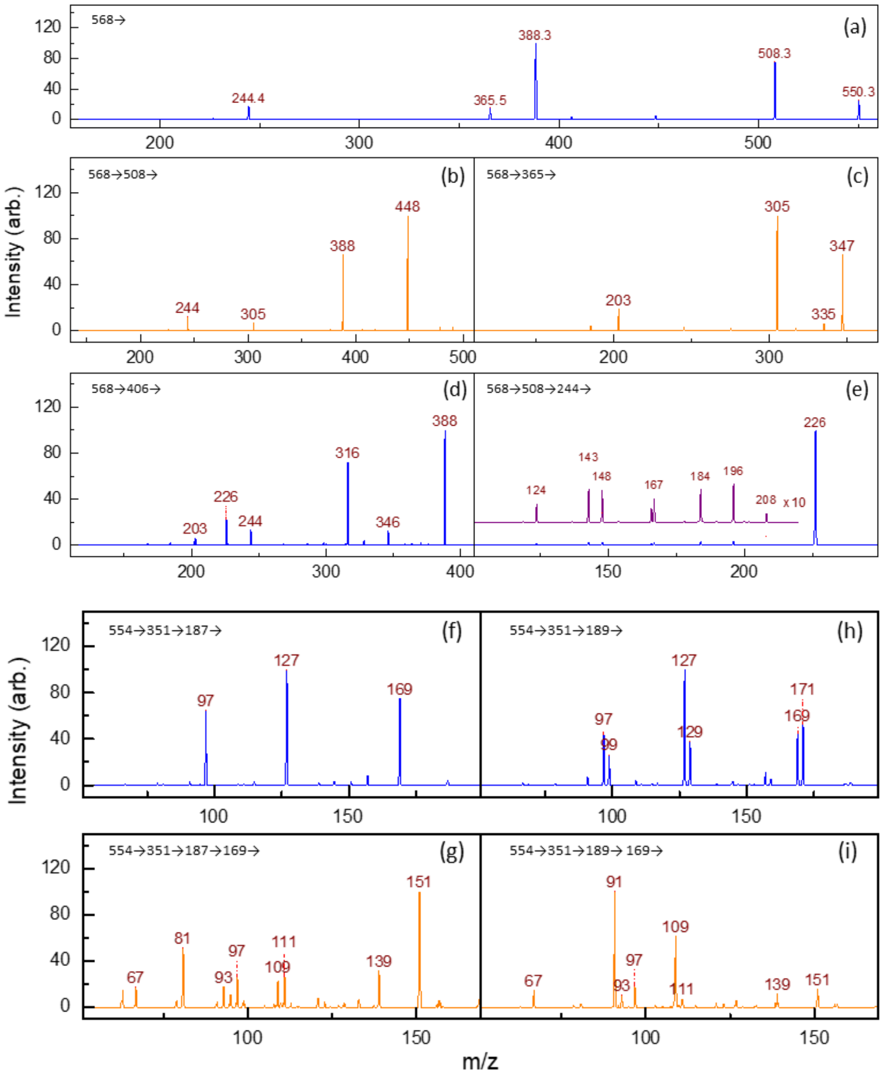

Figure S4.19. CID spectra of trisaccharide $(Hex)_2$HexNAc sodium adduct (a)-(e) and ^{18}O labelled (at O1 of the sugar at reducing end) lithium adduct (f)-(i) at retention time 32.1 min in Figure 1(e). Fragment ion *m/z* 508 in (a) indicates the trisaccharide is linear with a linkage of 1→4 at the reducing end or branched with 1→6 and 1→4 linkages at the reducing end. Ion *m/z* 388 in (b) indicates the trisaccharide is linear. Ion *m/z* 305 in (c) and ion *m/z* 316 in (d) indicate the linkages of Hex-Hex and HexNAc-Hex are 1→4 and 1→3, respectively. Comparing the CID spectra of (e), (f) and (g), and (h) and (i) to the CID spectra in monosaccharide database (Figure S2 suggests that HexNAc, hexose at center, and hexose at reducing end are GalNAc, β-Gal (similarity scores are β-Glc: 57, β-Gal: 87, α-Man: 54), and Glc (similarity scores are Glc: 93, Gal: 90, Man: 80), respectively. Fragment ion *m/z* 305 in (c) and *m/z* 316 in (d) suggests the linkages at reducing end and nonreducing end are 1→4 and 1→3, respectively. Comparing (d) to disaccharide database GalNAc-(1→3)-Gal in Figure S2.6 suggests the linkage is α-1→3. Consequently, the trisaccharide is determined to be α-GalNAc-(1→3)-β-Gal-(1→4)-Glc.

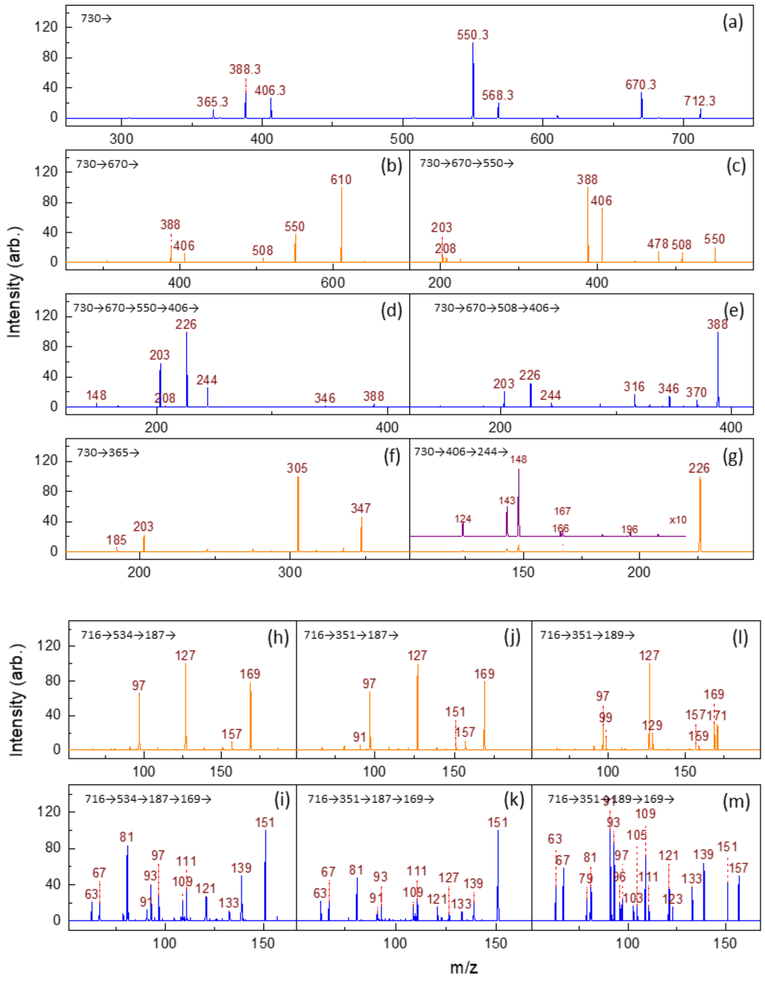

Figure S4.20. CID spectra of tetrasaccharide (Hex)$_3$HexNAc sodium adduct (a)-(g) and ^{18}O labelled (at O1 of the sugar at reducing end) lithium adduct (h)-(m) at retention time 21.6 and 22.4 min in Figure 3(a). Fragment ion *m/z* 670 in (a) indicates the sugar at the reducing end has a 1→4 linkage or (1→6, 1→4) linkages. Ion *m/z* 550 in (b) indicates the sugar at the reducing end is not branched. Ion *m/z* 406 in (c) indicates the three sugars not at the reducing end is linear. Ion *m/z* 508 in (b) indicates the sugar at the nonreducing end is a hexose. Ion *m/z* 406 in (c) indicates the sugar next to the sugar at the nonreducing end is a HexNAc. Comparing the CID spectra of (g), (h) and (i), (j) and (k), and (l) and (m) to the CID spectra in monosaccharide database (Figure S2) suggests that the hexose at nonreducing end, HexNAc, hexose at center, and the hexose at the reducing end are β-Gal (similarity scores are β-Glc: 55, β-Gal: 86, α-Man: 48), GlcNAc, β-Gal (similarity scores are β-Glc: 50, β-Gal: 81, α-Man: 50), and Glc (similarity scores are Glc: 77, Gal: 57, α-Man: 69), respectively. No fragment ion *m/z* 305 (loss of m=101) in (d) indicates the linkage at the nonreducing end is 1→3. Comparing (e) to disaccharide database GlcNAc-(1→3)-Gal in Figure S2.6 indicates the linkage is β-1→3. Consequently, the tetrasaccharide is β-Gal-(1→3)-β-GlcNAc-(1→3)-β-Gal-(1→4)-Glc.

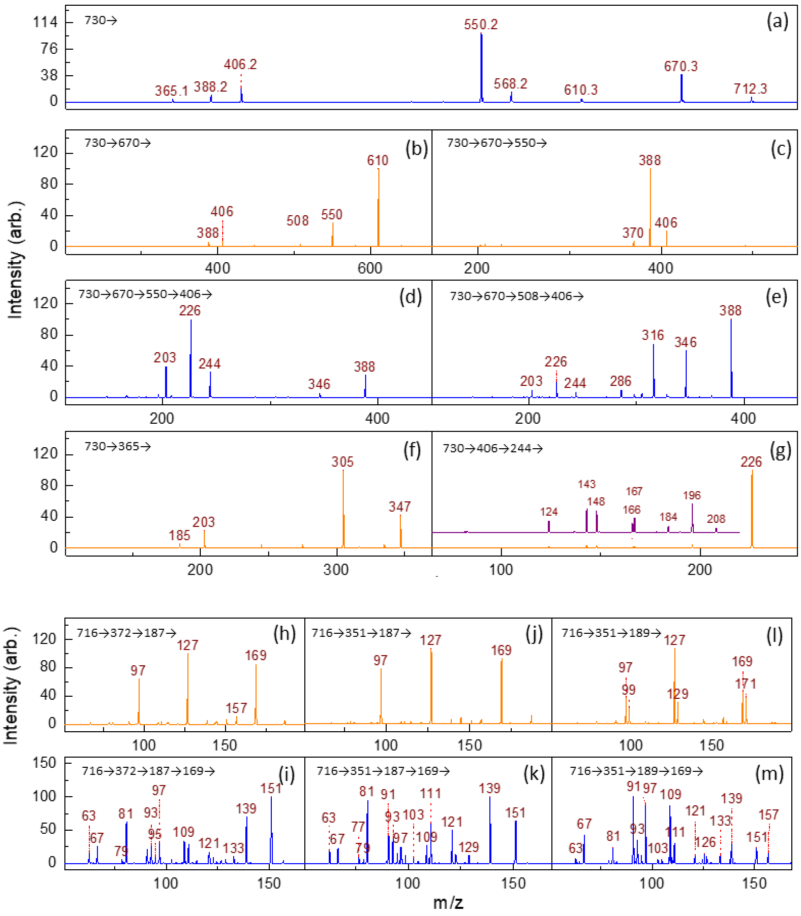

Figure S4.21. CID spectra of tetrasaccharide $(Hex)_3$HexNAc sodium adduct (a)-(g) and ^{18}O labelled (at O1 of the sugar at reducing end) lithium adduct (h)-(m) at retention time 15.8 and 17.0 min in Figure 3(b). Fragment ion *m/z* 670 in (a) indicates the sugar at the reducing end has a 1→4 linkage or (1→6, 1→4) linkages. Ion *m/z* 550 in (b) indicates the sugar at the reducing end is not branched, ion *m/z* 406 in (c) indicates the three sugars not at the reducing end is linear, therefore the tetrasaccharide is linear. Ion *m/z* 508 in (b) indicates the sugar at the nonreducing end is a hexose, ion *m/z* 406 in (c) indicates the sugar next to the sugar at the nonreducing end is a HexNAc, therefore the tetrasaccharide is Hex-HexNAc-Hex-Hex. Comparing the CID spectra of (g), (h) and (i), (j) and (k), and (l) and (m) to the CID spectra in monosaccharide database Figure S2 suggests that the hexose at nonreducing end, HexNAc, hexose at center, and the hexose at the reducing end are β-Gal (similarity scores are β-Glc: 80, β-Gal: 89, α-Man: 49), GalNAc, β-Gal (similarity scores are β-Glc: 67, β-Gal: 91, α-Man: 36), and Glc (similarity scores are Glc: 92, Gal: 79, α-Man: 78), respectively. No fragment ion *m/z* 305 (loss of m=101) in (d) indicates the linkage at the nonreducing end is 1→3. Comparing (e) to disaccharide database GalNAc-(1→3)-Gal in Figure S2.6 indicates the linkage is α-1→3. Consequently, the tetrasaccharide is β-Gal-(1→3)-α-GalNAc-(1→3)-β-Gal-(1→4)-Glc.

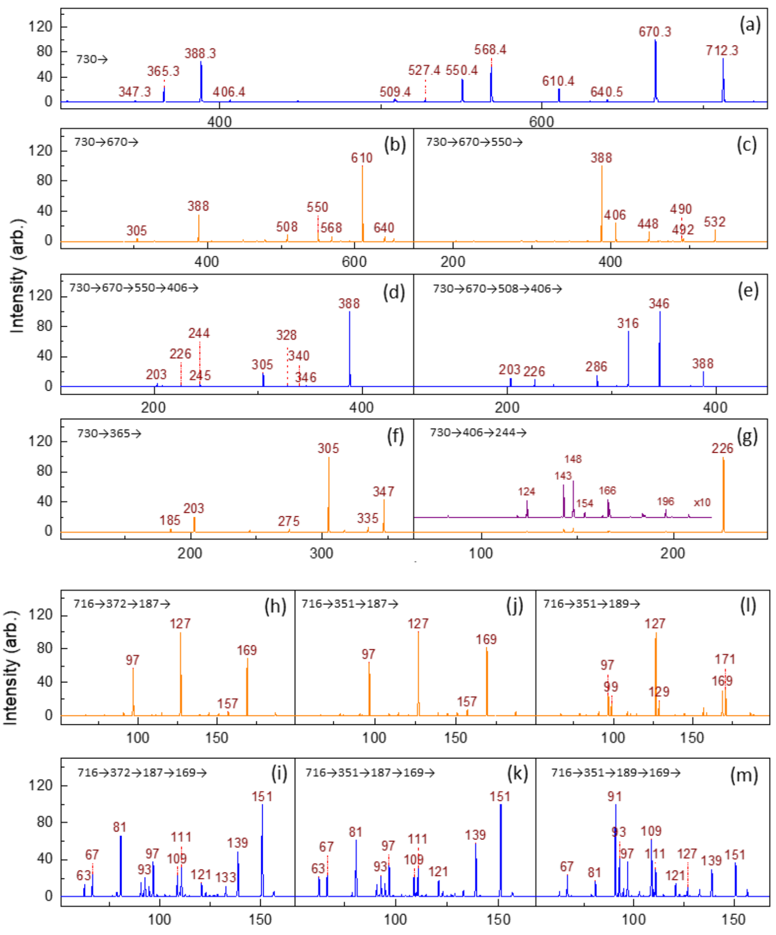

Figure S4.22. CID spectra of tetrasaccharide $(Hex)_3HexNAc$ sodium adduct (a)-(g) and ^{18}O labelled (at O1 of the sugar at reducing end) lithium adduct (h)-(m) at retention time 17.8 and 19.3 min in Figure 3(b). Fragment ion *m/z* 670 in (a) indicates the sugar at the reducing end has a 1→4 linkage or (1→6, 1→4) linkages. Ion *m/z* 550 in (b) indicates the sugar at the reducing end is not branched. Ion *m/z* 406 in (c) indicates the three sugars not at the reducing end is linear. Ion *m/z* 508 in (b) indicates the sugar at the nonreducing end is a hexose. Ion *m/z* 406 in (c) indicates the sugar next to the sugar at the nonreducing end is a HexNAc. Comparing the CID spectra of (g), (h) and (i), (j) and (k), and (l) and (m) to the CID spectra in monosaccharide database (Figure S2) suggests that the hexose at nonreducing end, HexNAc, hexose at center, and the hexose at the reducing end are β-Gal (similarity scores are β-Glc: 64, β-Gal: 95, α-Man: 46), GlcNAc, β-Gal (similarity scores are β-Glc: 66, β-Gal: 97, α-Man: 42), and Glc (similarity scores are Glc: 95, Gal: 76, α-Man: 83), respectively. Fragment ions *m/z* 305 and 328 in (d) indicate the linkage is 1→4 at nonreducing end. Fragment ions *m/z* 346, 316, and 286 in (e) indicates the linkages at center is 1→6. Comparing (e) to disaccharide α-GlcNAc-(1→6)-Gal and β-GlcNAc-(1→6)-Gal in Figure S2.6 indicates it is β-GlcNAc-(1→6)-Gal. Consequently, the tetrasaccharide is determined to be β-Gal-(1→4)-β-GlcNAc-(1→6)-β-Gal-(1→4)-Glc.

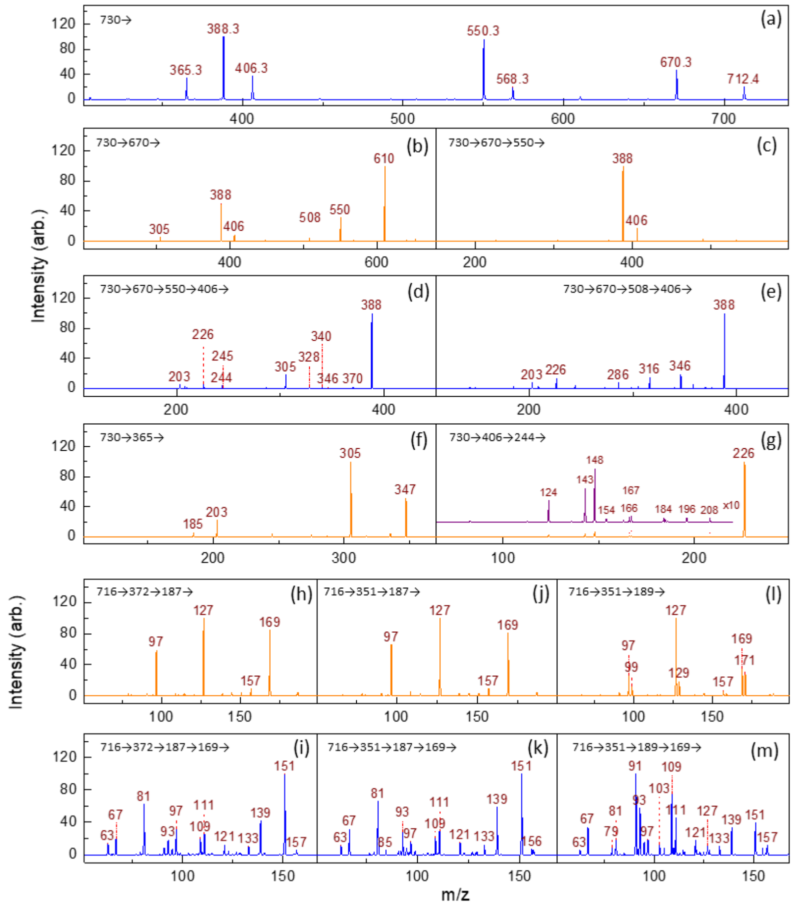

Figure S4.23. CID spectra of tetrasaccharide $(Hex)_3HexNAc$ sodium adduct (a)-(g) and ^{18}O labelled (at O1 of the sugar at reducing end) lithium adduct (h)-(m) at retention time 22.4 and 23.0 min in Figure 3(b). Fragment ion *m/z* 670 in (a) indicates the sugar at the reducing end has a 1→4 linkage or (1→6, 1→4) linkages. Ion *m/z* 550 in (b) indicates the sugar at the reducing end is not branched. Ion *m/z* 406 in (c) indicates the three sugars not at the reducing end is linear. Ion *m/z* 508 in (b) indicates the sugar at the nonreducing end is a hexose. Ion *m/z* 406 in (c) indicates the sugar next to the sugar at the nonreducing end is a HexNAc. Comparing the CID spectra of (g), (h) and (i), (j) and (k), and (l) and (m) to the CID spectra in monosaccharide database in Figure S2 suggests the hexose at nonreducing end, HexNAc, hexose at center, and the hexose at reducing end are β-Gal (similarity scores are β-Glc: 61, β-Gal: 92, α-Man: 46), GlcNAc, β-Gal (similarity scores are β-Glc: 63, β-Gal: 94, α-Man: 46), and Glc (similarity scores are Glc: 91, Gal: 69, α-Man: 86), respectively. Ions *m/z* 305 and 328 in (d) indicate the linkage of the disaccharide at nonreducing end is 1→4; no ion m/z 305 in (e) suggests the linkage of the disaccharide at center is 1→3. Comparing (e) to disaccharide database α-GlcNAc-(1→3)-Gal and β-GlcNAc-(1→3)-Gal in Figure S2.6 indicates the linkages at center is β-1→3. Consequently, the tetrasaccharide is β-Gal-(1→4)-β-GlcNAc-(1→3)-β-Gal-(1→4)-Glc.

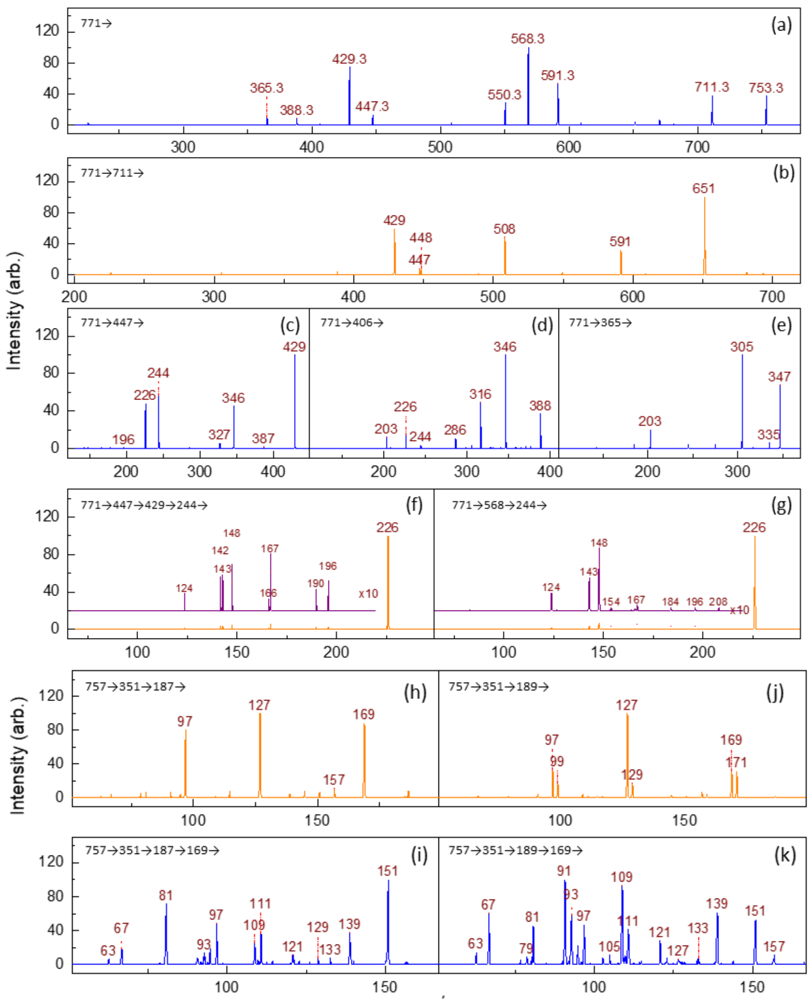

Figure S4.24. CID spectra of tetrasaccharide $(Hex)_2(HexNAc)_2$ sodium adduct (a)-(g) and ^{18}O labelled (at O1 of the sugar at reducing end) lithium adduct (h)-(k) at retention time 16.9 and 17.1 min in Figure 3(c). Fragment ion *m/z* 711 in (a) indicates the sugar at the reducing end has a 1→4 linkage or (1→6, 1→4) linkages. Ions *m/z* 365 and 447 in (a) and ion *m/z* 591 in (b) indicates the tetrasaccharide is linear with structure HexNAc-HexNAc-Hex-Hex. Comparing the CID spectra of (f), (g), (h) and (i), and (j) and (k) to the CID spectra in monosaccharide database in Figure S2 suggests that they are GalNAc, GlcNAc, β-Gal (similarity scores are β-Glc: 57, β-Gal: 88, α-Man: 54), and Glc (similarity scores are Glc: 82, Gal: 64, Man: 79), respectively. Ion *m/z* 346 in (c) indicate the linkage of the disaccharide at nonreducing end is 1→4 or 1→6. Comparing (c) to disaccharide database GalNAc-(1→6)-GlcNAc and GalNAc-(1→4)-GlcNAc in Figure S2.7 indicates the linkage is β-1→4. Ions *m/z* 346, 316, and 286 in (d) suggest the linkage of the disaccharide at center is 1→6 linkage. Comparing (d) to disaccharide database α-GlcNAc-(1→6)-Gal and β-GlcNAc-(1→6)-Gal in Figure S2.6 indicates the linkage at center is β-1→6. Consequently, the structure is β-GalNAc-(1→4)-β-GlcNAc-(1→6)-β-Gal-(1→4)-Glc.

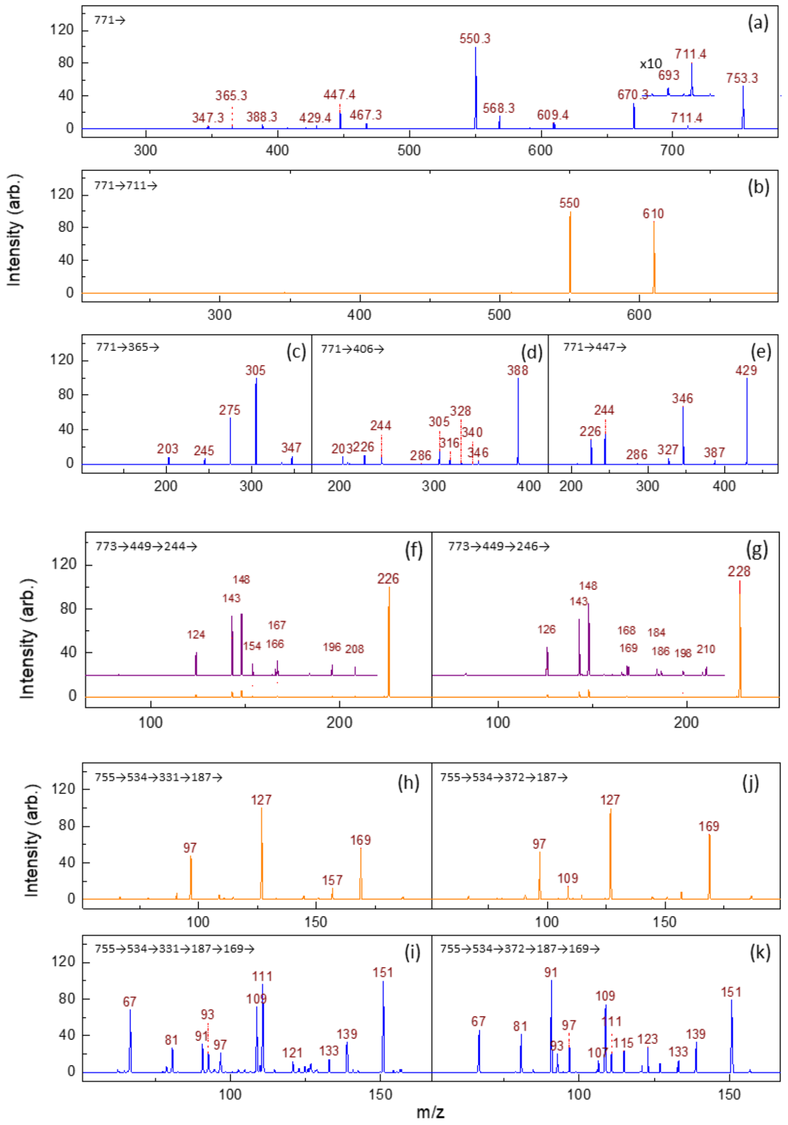

Figure S4.25. CID spectra of tetrasaccharide $(Hex)_2(HexNAc)_2$ sodium adduct (a)-(e), ^{18}O labelled (at O1 of the sugar at reducing end) sodium adduct [(f) and (g)], and lithium adduct (h)-(k), at retention time 17.4 and 22.4 min in Figure 3(c). Fragment ion *m/z* 670 and 693 in (a) indicates the sugar at the reducing end is HexNAc and has a 1→4 linkage. Ions *m/z* 365 and 447 in (a) and ion *m/z* 550 in (b) indicates the tetrasaccharide is linear Hex-Hex-HexNAc-HexNAc. Comparing (f), (g), (h) and (i), and (j) and (k) to monosaccharide database in Figure S2 suggests they are GlcNAc, GlcNAc, α-Man (similarity scores are β-Glc: 63, β-Gal: 50, α-Man: 84). Ions *m/z* 305, 275, and 245 in (c) indicate the linkage of the disaccharide at the nonreducing end is 1→6; ions *m/z* 305 and 328 the linkage of the disaccharide at center is 1→4. Comparing (d), and (e) to disaccharide database GlcNAc-(1→4)-GlcNAc in Figure S2.7 suggests the linkages at reducing end is β-1→4. The rest of the structure was determined by comparing the retention time of α-Man-(1→6)-β-Man-(1→4)-β-GlcNAc-(1→4)-GlcNAc in the chromatogram of

HPLC in Figure S3. Consequently, the tetrasaccharide is α-Man-(1→6)-β-Man-(1→4)-β-GlcNAc-(1→4)-GlcNAc.

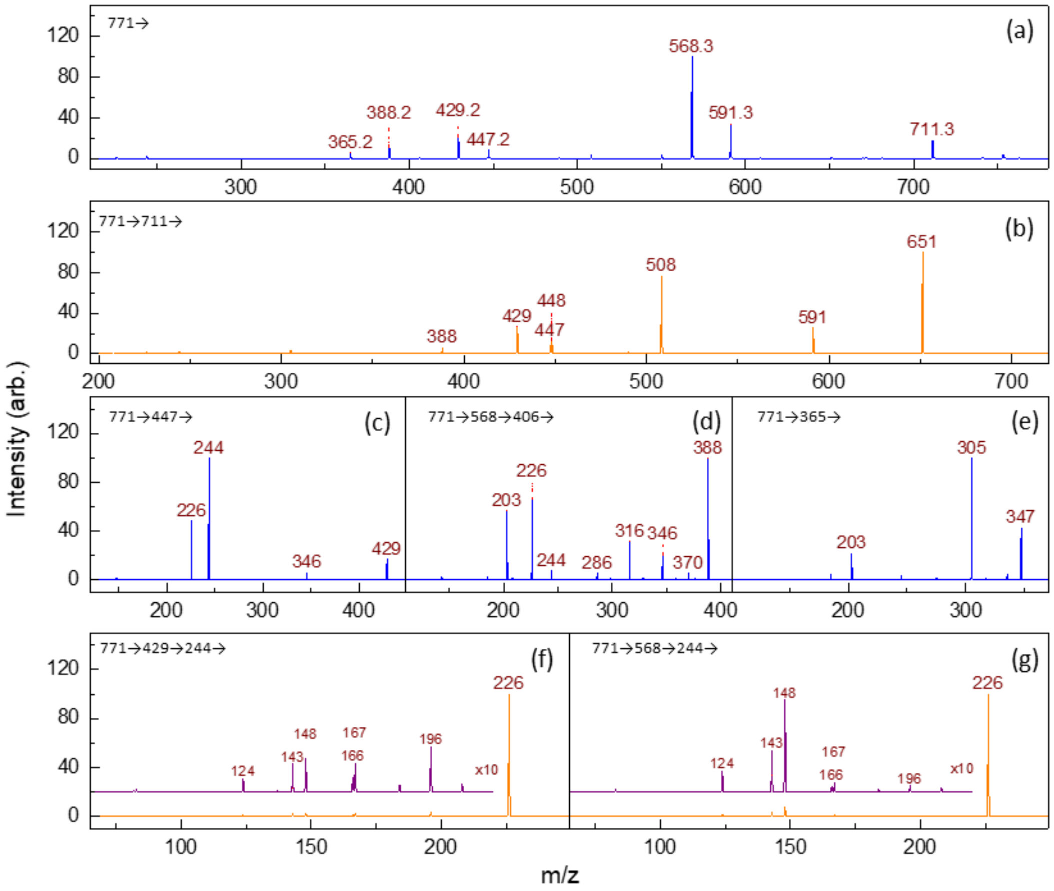

Figure S4.26. CID spectra of tetrasaccharide $(Hex)_2(HexNAc)_2$ sodium adduct at retention time 16.9 min (from the retention time 14.5 min of amdie-80 column) in Figure 3(d). Fragment ion *m/z* 711 in (a) indicates the sugar at the reducing end has a 1→4 linkage or (1→6, 1→4) linkages. Ions *m/z* 365 and 447 in (a) and ion *m/z* 591 in (b) indicates the tetrasaccharide is linear with structure HexNAc-HexNAc-Hex-Hex. Comparing the CID spectra of (f), and (g) to the CID spectra in monosaccharide database in Figure S2 suggests that they are GalNAc and GlcNAc, respectively. Comparing (c), (d), and (e) to disaccharide database indicates the linkages at nonreducing end, center, and reducing end are 1→3, 1→3, and β-1→4, respectively. The rest of the structures cannot be determined due to the low abundance of this tetrasaccharide in milk. The tetrasaccharide is determined to be GalNAc-(1→3)-GlcNAc-(1→3)-β-Hex-(1→4)-Hex.

5. NMR spectra of home-synthesized disaccharides and oligosaccharides

D-Gal-*β*-(1→4)-(D-Gal-*β*-(1→2))-D-Glc

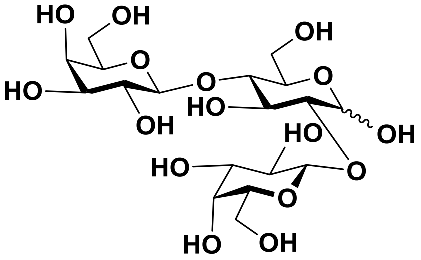

^{1}H NMR (600 MHz, D_2O): δ 5.41 (d, J = 3.7 Hz, 1H), 4.53 (d, J = 7.8 Hz, 1H), 4.42 (t, J = 8.3 Hz, 2H), 4.00 – 3.59 (m, 48H), 3.58 – 3.47 (m, 6H), 3.47 – 3.40 (m, 1H).

^{13}C NMR (150 MHz, D_2O): δ 104.4, 103.1, 102.8, 102.83, 102.79, 102.7, 96.4, 95.7, 92.2, 91.7, 91.6, 80.6, 80.1, 78.4, 78.2, 77.7, 75.3, 75.29, 75.2, 75.1, 75.0, 74.96, 74.8, 74.7, 74.6, 74.3, 73.7, 72.7, 72.68, 72.6, 72.56, 72.5, 72.4, 71.8, 71.3, 71.28, 71.2, 71.1, 70.99, 70.9, 70.9, 70.8, 70.7, 70.5, 70.4, 70.0, 69.9, 69.6, 69.5, 69.4, 69.3, 69.2, 69.1, 68.9, 68.6, 68.5, 68.49, 68.3, 61.1, 61.0, 61.0, 60.98, 60.92, 60.9, 60.0, 59.8.

D-Gal-*β*-(1→4)-D-Glc-*β*-(1→4)-D-Glc

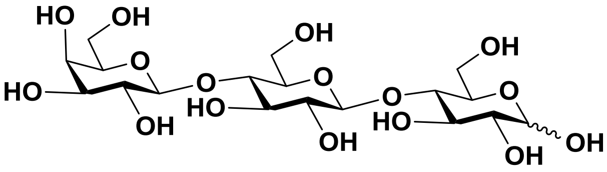

^{1}H NMR (600 MHz, D_2O): δ 5.13 (d, J = 3.8 Hz, *α*, 1H), 4.57 (d, J = 8.0 Hz, *β*, 1H), 4.44 (d, J = 7.9 Hz, 2H), 4.36 (d, J = 7.8 Hz, 2H), 3.92 – 3.81 (m, 9H), 3.81 (dd, J = 5.0, 2.4 Hz, 1H), 3.79 – 3.76 (m, 2H), 3.76 – 3.61 (m, 16H), 3.60 – 3.50 (m, 16H), 3.49 (d, J = 3.6 Hz, 1H), 3.48 – 3.42 (m, 3H), 3.42 – 3.37 (m, 1H), 3.32 (ddd, J = 9.8, 7.3, 1.6 Hz, 1H), 3.27 (ddd, J = 9.1, 7.9, 4.6 Hz, 2H), 3.24 – 3.20 (m, 1H), 3.19 (t, J = 8.5 Hz, 1H).

^{13}C NMR (150 MHz, D_2O): δ 102.9, 102.5, 102.5, 102.3, 102.3, 96.4, 95.8, 95.7, 92.2, 92.0, 91.8, 78.7, 78.6, 78.5, 78.4, 78.1, 78.0, 75.9, 75.9, 75.7, 75.4, 75.3, 75.1, 74.8, 74.76, 74.2, 74.2, 74.1, 73.8, 73.1, 72.8, 72.8, 72.7, 72.5, 71.8, 71.4, 71.38, 71.3, 71.26, 71.2, 70.9, 70.1, 69.5, 69.4, 69.4, 69.3, 68.7, 68.5, 68.3, 61.5, 61.0, 60.9, 60.7, 60.5, 60.0, 59.9, 59.9, 59.8, 58.6.

D-Gal-*β*-(1→4)-(D-Glc-*α*-(1→2))-D-Glc

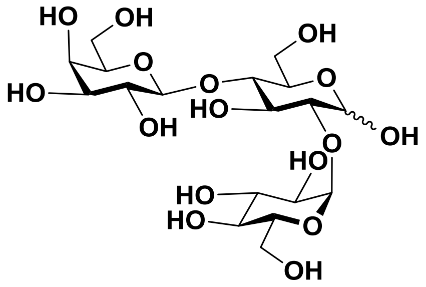

^{1}H NMR (600 MHz, D_2O): δ 5.43 (d, J = 3.6 Hz, 1H), 5.35 (d, J = 3.9 Hz, 1H), 5.09 (d, J = 3.8 Hz, 1H), 4.83 – 4.81 (m, 1H), 4.45 (t, J = 7.5 Hz, 2H), 4.07 (dd, J = 10.2, 3.2 Hz, 1H), 4.00 – 3.89 (m, 7H), 3.89 – 3.82 (m, 3H), 3.82 – 3.68 (m, 20H), 3.68 – 3.63 (m, 4H), 3.60 – 3.57 (m, 1H), 3.57 – 3.50 (m, 5H), 3.48 – 3.39 (m, 4H).

^{13}C NMR (150 MHz, D_2O): δ 102.9, 102.8, 97.79, 96.4, 96.0, 89.1, 78.5, 78.2, 78.17, 75.5, 75.3, 74.6, 73.1, 72.3, 72.74, 72.7, 72.5, 71.7, 71.5, 71.4, 71.39, 70.9, 69.9, 69.8, 69.3, 69.2, 68.6, 68.5, 61.1, 61.0, 60.2, 60.1, 60.0, 59.9, 53.8.

D-Gal-*β*-(1→4)-(D-GalNAc-*β*-(1→2))-D-Glc

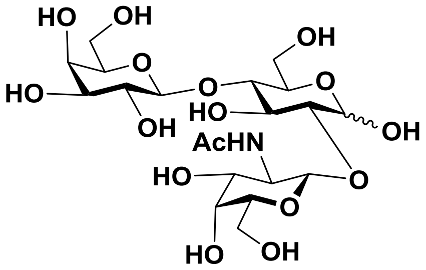

^{1}H NMR (600 MHz, D_2O): δ 5.13 (d, *J* = 3.0 Hz, 1H), 4.58 (dd, *J* = 8.0, 1.3 Hz, 1H), 4.55 (dd, *J* = 8.5, 1.5 Hz, 1H), 4.36 (dd, *J* = 7.8, 1.2 Hz, 2H), 4.07 – 4.00 (m, 2H), 3.93 – 3.81 (m, 11H), 3.60 – 3.55 (m, 8H), 3.55 – 3.43 (m, 7H), 3.20 (ddd, *J* = 9.3, 8.1, 1.2 Hz, 1H), 1.98 – 1.94 (m, 6H).

^{13}C NMR (150 MHz, D_2O): δ 175.19, 174.96, 174.69, 103.27, 102.88, 102.86, 102.81, 96.41, 95.86, 95.72, 95.37, 92.04, 91.78, 91.50, 90.96, 78.39, 78.25, 75.90, 75.70, 75.32, 75.28, 75.15, 75.11, 74.94, 74.77, 74.73, 74.33, 74.08, 73.77, 72.48, 71.38, 71.11, 71.08, 70.99, 70.93, 70.83, 70.70, 70.50, 70.08, 69.91, 69.59, 69.55, 68.54, 68.52, 67.81, 67.74, 67.34, 61.19, 61.10, 61.02, 61.01, 60.97, 60.94, 60.69, 60.53, 60.04, 59.91, 58.57, 53.61, 52.55, 52.35, 50.22, 22.25, 22.18, 21.93.

D-GlcNHAc-*β*-(1→6)-D-Gal-*β*-(1→4)-D-Glc

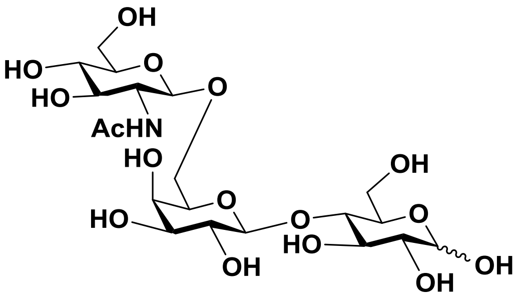

^{1}H NMR (600 MHz, D$_2$O): δ 5.14 (d, *J* = 3.8 Hz, 1H), 5.11 (d, *J* = 3.6 Hz, 1H), 4.58 (d, *J* = 7.9 Hz, 1H), 4.53 (dd, *J* = 8.5, 2.5 Hz, 1H), 4.49 (d, *J* = 6.6 Hz, 1H), 4.44 – 4.41 (m, 1H), 4.36 (dd, *J* = 8.1, 6.1 Hz, 2H), 3.91 – 3.42 (m, 79H), 3.38 (tdd, *J* = 12.4, 10.0, 6.7 Hz, 8H), 3.24 – 3.18 (m, 1H), 1.99 – 1.94 (m, 12H).

^{13}C NMR (150 MHz, D$_2$O): δ 174.7, 174.6, 174.53, 174.50, 174.4, 103.0, 102.99, 102.9, 102.89, 102.86, 101.6, 101.5, 101.43, 101.4, 101.1, 101.0, 98.2, 96.7, 96.4, 95.9, 95.7, 95.7, 94.9, 91.8, 90.8, 79.5, 79.2, 78.9, 78.7, 78.4, 78.4, 78.3, 77.4, 75.9, 75.9 75.8, 75.7, 75.3, 75.1, 74.8, 74.7, 74.3, 73.9, 73.8, 73.78, 73.7, 73.5, 73.3, 72.6, 72.5, 72.4, 72.3, 72.2, 72.1, 71.8, 71.5, 71.4, 71.36, 71.2, 71.1, 71.08, 70.9, 70.93, 70.87, 70.8, 70.7, 70.6, 70.5, 70.1, 70.1, 70.0, 69.9, 69.89, 69.8, 69.79, 69.6, 69.5, 69.4, 69.38, 69.2, 68.8, 68.6, 68.5, 68.4, 68.37, 68.2, 68.1, 67.8, 67.0, 62.6, 62.5, 62.4, 62.0, 61.9, 61.5, 61.45, 61.4, 61.3, 61.0, 60.7, 60.7, 60.6, 60.5, 60.5, 60.1, 60.0, 59.9, 59.9, 58.6, 56.6, 55.6, 55.5, 55.4, 54.0, 53.7, 27.8, 27.8, 22.3, 22.3, 22.2, 22.1, 22.1, 22.09, 21.9, 21.8, 16.0, 15.5.
